# Effects of Core Strength Training on Maximal Trunk Muscle Strength and Cycling Economy in Female Mountain Bikers

**DOI:** 10.1186/s40798-026-00996-0

**Published:** 2026-03-09

**Authors:** Roland Blechschmied, Jana Strahler, Urs Granacher

**Affiliations:** 1 Department of Sport and Sport Science, Exercise and Human Movement Science, University of Freiburg, Sandfangweg 4, 79102 Freiburg, Germany; 2 Department of Sport and Sport Science, Sport Psychology, University of Freiburg, Freiburg, Germany

**Keywords:** Adolescent, Cardiorespiratory fitness, Muscle strength, Resistance training, Youth sports

## Abstract

**Background:**

In cross-country mountain biking (MTB), performance is primarily determined by aerobic capacity. However, shortened race durations from 150 to 80 min increased the relevance of anaerobic capacity, muscle strength, and power. Adequate levels of trunk muscle strength (TMS) enable force transfer between lower and upper extremities during starts, inclines, and finishes. Cycling economy (CE) may benefit from TMS training. This study examined the effects of TMS training on TMS, TMS endurance, and CE in MTB athletes.

**Methods:**

Twenty-four trained or highly-trained female (Tier 2–3) MTB athletes aged 14–22 years were pair-matched by age and randomly assigned to a TMS group or an active control. Control exercised lower limb but not trunk muscles. Over eight weeks during the off-season, both groups completed three weekly 30-min sessions. Pre-, post training, assessments included maximal isometric TMS, lateral trunk endurance, and CE. Hormonal measures were included to account for menstrual cycle–related variability. CE was assessed during an MTB race course treadmill simulation via physiological (O₂/CO₂·min⁻¹·kg⁻¹) and CE-related mechanical parameters.

**Results:**

TMS training improved maximal trunk flexor (*p*<0.001, d = 1.1), extensor strength (*p*<0.001, d = 1.7), and lateral TMS endurance (*p*=0.03, d = 0.74), compared with controls. Lateral bike displacement was reduced in the TMS group (*p*=0.001, d = 0.8). Physiological CE parameters showed no between-group differences. Hormonal measures did not moderate effects (*p*>0.05).

**Conclusions:**

Eight weeks of global TMS training improved maximal isometric trunk flexor, extensor strength and lateral bike displacement in female MTB athletes. These findings suggest that off-season TMS training may improve MTB performance.

**Supplementary Information:**

The online version contains supplementary material available at 10.1186/s40798-026-00996-0.

## Key Points

- This study found improvements in maximal isometric trunk muscle flexor and extensor strength, lateral trunk muscle endurance, and mechanical factors affecting cycling economy, reflected in reduced lateral bike displacement after 8 weeks of trunk muscle strength training, compared to traditional strength training that did not target the trunk muscles.
- Trunk muscle strength training is an effective training modality that can be implemented during the off-season to improve performance related factors in female mountain bikers.

**Supplementary Information:**

The online version contains supplementary material available at 10.1186/s40798-026-00996-0.

## Background

In competitive cross-country mountain biking (MTB), performance is shaped by a complex interplay of aerobic capacity, anaerobic power, and biomechanical efficiency. The total energy turnover is primarily determined by aerobic capacity [1], with approximately 63% of the energy being provided through the aerobic metabolism and 37% through anaerobic pathways [2]. In race-decisive moments such as mass starts, climbs and final sprints, the anaerobic-lactic energy system appears to be an additional key performance determinant, due to large energy demands in short periods of time [1–3]. Since the official reduction of cross-country race durations from over 150 min to 80 min in 2007, the importance of the anaerobic metabolism for race performance has further increased [4]. Physiological cycling economy (CE), reflected in oxygen (O₂) consumption and exhaled carbon dioxide (CO₂) at a given power output, is a key metric in cycling performance [1, 5]. There is evidence that CE strongly correlates with a higher power output in Watts at the individual anaerobic threshold [6, 7]. An important factor influencing CE is the mechanical efficiency of the pedal strokes [8]. Mornieux and colleagues [8] reported that an effective pedaling technique is characterized by a higher percentage of forward propulsion primarily produced from lower limb muscles. Cross-country races are characterized by technical challenges, including jumps and steep inclines, which require sufficient levels of maximal muscle strength and power, as well as adequate bicycle handling skills while mastering technically challenging race courses [1]. During these race situations, arm and leg muscles act as “shock absorbers,” compensating for ground impacts and perturbations during pedaling [1, 9]. In mountain biking, trunk muscles play an important role within the kinetic muscle chain as they serve as a link between the lower and upper body, enabling the efficient transfer of forces generated by the leg muscles for bike propulsion [1, 10]. In other words, proximal stability of the trunk muscles provides the foundation for distal limb mobility and performance [1, 10, 11].

In the context of trunk muscles, Bergmark [12] distinguished between local and global trunk muscles. While the deep lying local trunk muscles have attachment to the lumbar vertebrae, the global or superficial trunk muscles are those with attachments to the hips and pelvis and therefore influence spinal orientation and the control of external forces on the spine [12, 13]. While the local trunk muscles provide trunk stability at static or slower movements, therefore enabling trunk muscle strength (TMS) endurance, the global trunk muscles enable high torque generation which facilitate force transfer throughout the body [14]. Different research groups have previously examined the role of trunk muscles with metabolic costs in road cycling [15–17]. For instance, McDaniel and colleagues [16] reported that a mechanical trunk stabilizer in the form of a padded metal trunk support device attached to the saddle resulted in significantly reduced metabolic costs (higher CE), especially at higher pedaling forces [16]. Miller and colleagues compared the metabolic costs when cycling on a stable bike ergometer versus an unstable cycling trainer roller in highly-trained male cyclists. Findings indicated significantly higher metabolic costs when riding on the cycling trainer roller which is most likely due to higher trunk stability demands on the unstable training device [15].

Unstable surfaces on trails (rocks, tree roots) are frequently encountered in MTB and impact on the bike and ultimately the rider [1]. Previously, researchers have demonstrated that improved TMS positively affects movement economy and performance across multiple sports, including canoe sprinting, handball, and soccer [5, 18, 19]. Hibbs et al. [14] concluded that in order to successfully induce adaptations in TMS, heavy resistance strength training focusing on the global trunk muscles seems to have larger performance enhancing effects compared with local trunk muscle training [12, 20, 21]. Despite its potential to unlock additional adaptive reserves in MTB, TMS training has received little attention in MTB so far [19]. As in many other sports, the “gender data gap” is also prevalent in MTB with only few available studies on female athletes [22]. However, findings from training studies with male MTB riders cannot be transferred to female athletes (although adaptations are similar) due to significant hormonal, anatomical, and biomechanical differences [23, 24]. In particular, fluctuations in steroid hormones such as estrogen, progesterone and cortisol throughout the menstrual cycle, affect muscle strength, metabolism, endurance, and recovery [25–27]. Consequently, these fluctuations may influence physiological responses to training in female athletes. Given these findings, future research should systematically account for these hormonal variations to better understand female performance development and how female athletes respond to training stimuli.

Thus, the main objective of this study was to examine the effects of global TMS training versus traditional strength training (active control) on maximal TMS, TMS endurance, CE and body composition in female MTB athletes. With reference to the relevant literature [5, 10, 14], we hypothesized that global TMS training leads to significantly larger performance improvements. A secondary objective was to analyze the potential role of menstrual cycle related hormonal fluctuations on training-induced outcomes [27].

## Methods

To test our hypothesis, adaptations following the training period were analyzed using a randomized matched-pairs intervention study design. In line with open science practices, this study’s protocol and analysis plan were pre-registered on the Open Science Framework (OSF). The pre-registration document is available at (10.17605/OSF.IO/TCY2S).

###  Participants

After the initial screening of 27 athletes, 24 trained or highly-trained females with Tier 2–3 training and performance calibre finally volunteered to participate and were eligible to be included in this study (Table 1) [28]. MTB athletes were recruited from regional or national squads.

**Table 1:** Baseline characteristics of the study athletes according to group allocation

Variable	Active control	TMS	*P*
	(*n* = 12)	(*n* = 12)	
Age (years)	17.4 ± 3.1	17.2 ± 4.0	0.91
Body height (cm)	166.4 ± 6.2	164.7 ± 5.4	0.49
Body mass (kg)	58.9 ± 8.5	55.5 ± 5.8	0.27
MTB experience (years)	8.4 ± 4.0	9.2 ± 1.5	0.53
Body-mass-index (BMI)	21.2 ± 1.2	20.4 ± 0.9	0.87

Values are means ± standard deviations (SDs). Age = age in years; Body height = body height in centimeters; Body mass = body mass in kilograms; MTB experience = years of mountain biking experience; BMI = body mass index

**Fig. 1:**
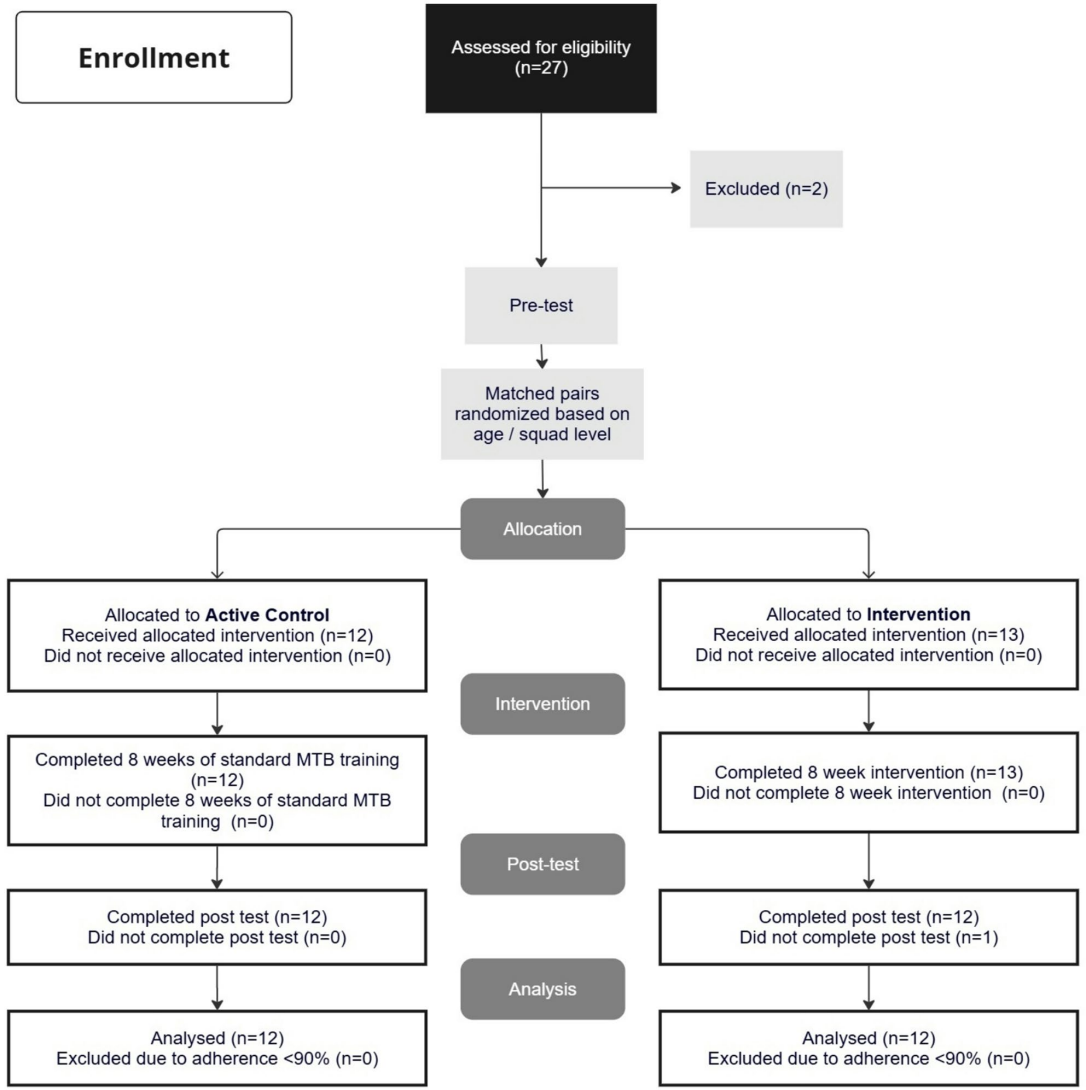
Flow chart of the progress through the phases of the study according to the CONSORT statements

The required sample size was determined via an a priori power analysis, based on effect sizes reported in a related study employing a similar study design [5]. The reported effect size for CE (f = 0.5) was used in the calculation, with an alpha level of 0.01 and a statistical power of 0.9. It is important to note that, due to the lack of intervention studies specifically investigating global trunk muscle strength training in mountain bikers, the referenced study was selected as the most suitable proxy to estimate expected changes in cycling economy. analysis indicated that a minimum of *N* = 20 athletes would be required to detect statistically significant within- and between-group interactions. As we expected drop-outs over the eight-week intervention period due to injuries or illness, we opted for *N* = 25 participants. Athletes were assigned to a TMS (*n* = 12) or an active control (CON) (*n* = 13) in randomized but matched pairs according to age. Research randomizer software (http://www.randomizer.org/) was used. Inclusion criteria were > 5 years of MTB experience, absence of injury for at least six months prior to the start of the study, age 14–22 years, and a training and performance caliber of the highest regional performance or national level (Tier 2–3) (Fig. 1).

Table 1 shows the characteristics of the participating athletes who completed a minimum of 80% of the scheduled training sessions. All athletes and their legal representatives were informed about potential benefits and risks of the study prior to study participation. Written informed consent was obtained from the athletes and their legal representatives. Ethical approval was received from the ethics committee of the University hospital Freiburg, Germany (approval number: 24-1179-S2).

### Study Design

The TMS group completed an eight-week off-season MTB training with three weekly sessions, each lasting 30 min. The off-season phase in MTB typically emphasizes recovery, maintenance of general strength, and the development of basic aerobic endurance through low-intensity cycling. The global TMS training replaced the athletes’ traditional off-season resistance training during the intervention period. This approach was chosen to ensure comparable overall training volume between groups and to avoid confounding effects related to differences in total training load. Adding TMS training on top of the existing strength training program would likely have resulted in volume-driven adaptations rather than effects attributable to the specific training content. The off-season was selected to implement the TMS intervention, as this period allows for consistent training without interference from competition-induced fatigue or tapering strategies. The CON group maintained the regular “treatment as usual” off-season MTB training program consisting of three weekly 30 min upper- and lower-body strength training sessions without the performance of specific trunk exercises. Both groups conducted similar outdoor MTB training regimes on non-strength training days with two-to-three weekly sessions, depending on weather conditions. MTB specific training sessions lasted between one-two hours and were conducted at moderate intensity (< 75% of maximal heart rate). The study was scheduled during the German autumn / winter season. Training volumes were similar between the experimental groups.

### Study Protocol

Before testing, athletes’ lifestyle and nutritional habits were assessed using a combined questionnaire based on the Swiss Olympic Manual for Performance Diagnostics, the PAR-Q, and the DEGS questionnaire from the Robert Koch Institute (RKI) [29, 30]. The complete questionnaire is provided as supplementary material (supplementary material, appendix 1). Athletes were included if they used hormonal contraception or followed their natural menstrual cycle. Cycle regularity was documented in advance, although irregular cycles were not taken as an exclusion criterion. The menstrual cycle phase was determined using a questionnaire adapted from the cycle diary by Rohde and Dorn [26]. Pre- and post-tests were classified based on menstrual cycle phase (luteal or follicular). Severe menstrual symptoms or ovulation were noted as an exclusion criterion for testing on the respective days. In case of severe symptoms, athletes were asked to attend the test sessions on an alternative day.

#### Mountain Bike Specific Test

For MTB-specific testing, a 5 × 3 m treadmill was used (ForceLink B.V., Culembourg, Netherlands). The treadmill allowed a maximum speed of 45 km/h, an incline of up to 24%, and a decline of -4%, contributing to more ecologically valid and at the same time challenging test conditions. The treadmill protocol consisted of a replica of the altitude profile of the race course in Gedern, Germany, a regular venue of the German Women’s Cross-Country Championships. This twelve-minute course featured alternating climbs, descents and flat sections, providing a high-intensity challenge representative of MTB specific demands (supplementary material, appendix 2). To account for the rotational components typical for MTB courses, a slalom element was included on different sections of the treadmill protocol. For this purpose, a laser projected a sinusoidal curve onto the treadmill surface, which athletes had to follow as precisely as possible. Athletes were required to follow the protocol’s inclines, declines, and curves. The study supervisor visually inspected whether participants followed the laser indicating the turns. In case of deviation, a warning was issued. If the participant failed to follow the laser a second time, the test was terminated. All participating athletes were familiarized with cycling on the treadmill due to previous performance tests. The individual treadmill speed was determined based on prior performance data of each athlete. Treadmill speed was individualized for each athlete and was equal during pre and post-tests. The adjustment formula for individual treadmill speed was determined using an empirically derived linear model based on data from three athletes who were not part of the study cohort but competed at a comparable MTB performance level. Each of the three athletes repeatedly completed the MTB-specific treadmill protocol, and the maximal sustainable velocity was identified based on subjective exertion and heart rate responses. These empirically determined velocities were then related to the athletes’ functional threshold power, resulting in a linear regression model that was subsequently used to estimate individual treadmill speeds for all participants. Detailed calculations and individual adjustments are provided in the supplementary material (appendix 3). In brief, the functional threshold power (FTP) served as the basis to individualize the treadmill protocol. To standardize conditions, suspension settings as well as tire air pressure were noted upon the first test and replicated during post-tests (high- and low-speed compression, rebound clicks, psi in front and rear shock). Dropper post height and handlebar positions were further kept at the same setting during pre and post-tests. Individual crank lengths were programmed within the used cycling computer. Before the tests started, athletes were given a ten-minute familiarization and warm-up phase on the treadmill, practicing inclines, declines and the turning maneuvers.

### Test-Retest Reliability of the MTB Specific Test

The assessment of test-retest reliability was realized prior to the training study. For this purpose, ten randomly selected study participants with Tier 2–3 training and performance caliber (trained, highly-trained MTB athletes), but not the final study participants. None of the participants had prior experience riding a bicycle on a treadmill completed two laboratory visits within one week at similar day time to minimize diurnal performance fluctuations. A minimum of 24 h recovery was granted between tests. Prior to the test-re-test analysis, information were provided to standardize the sleep status, caffeine intake, and nutrition on both test days to reduce the potential impact of these variables. Parameter-specific results of the reliability study can be found in Table 2.

**Table 2:** Relative and absolute test retest reliability for the assessed parameters Coefficients of variation (CV), Pearson correlations and intraclass correlation coefficients (ICC)

Variable	CV	Pearson	ICC
Heart rate	0.05	0.93	0.93
Isometric maximal strength ventral	0.02	0.98	0.98
Isometric maximal strength dorsal	0.01	0.99	0.97
Lateral displacement	0.03	0.95	0.95
Cadence	0.10	0.75	0.89
Normalized power	0.13	0.99	0.99
Balance L/R	0.05	0.96	0.97
Torque effectiveness	0.09	0.93	0.95
Pedal smootheness	0.09	0.97	0.93

Balance L/R = balance left/right

### Anthropometrics and Body Composition

Anthropometric measurements, including stature and mass, were assessed using a calibrated stadiometer and digital scale (SECA 285, Hamburg, Germany). Athletes were measured in light clothing and without shoes, following standardized procedures to ensure accuracy and reliability. Body stature was recorded to the nearest 0.1 cm, and body mass to the nearest 0.1 kg. These measurements were used to calculate the body mass index (BMI) as body mass (kg) divided by height (m²).

Body composition was assessed using a bioelectrical impedance analysis system (SECA mBCA 515, Hamburg, Germany), measuring electrical resistance to estimate lean and fat mass. Athletes followed a standardized protocol including fasting for 6 h, avoiding exercise for twelve hours, and abstaining from alcohol and caffeine for 24 h to ensure measurement reliability and validity [31]. Electrodes were placed on the hand and foot, and calculations were based on validated hydration constants. This method has previously shown good reliability with a coefficient of variation (CV) < 2% [31].

### Physical Fitness Tests

#### Maximal Isometric Trunk Muscle Strength

Maximal isometric flexor and extensor TMS was tested using an electromechanical dynamometer in a setup as described by Rodriguez-Perea et al. [32]. Test-retest reliability is reported in Table 2. In accordance with the study of Rodriguez-Perea et al. [32], maximal isometric extensor and flexor TMS was measured in a seated position on a bench with the feet side by side and shoulder width apart. Sliding forward on the bench was not possible because the athletes were fixed with straps and belts (supplementary material, appendix 4). The seated posture was chosen to isolate trunk muscle activation, and to reduce iliopsoas muscle activity. The 90° angle relative to the thigh provides greater stability because the pelvis is fixed through straps during testing [32]. The athletes wore a harness attached to a piezoelectric force transducer (type 9311B; Kistler^®^, Winterthur, Switzerland) with a metal lock and the force transducer was attached to the measurement frame (supplementary material, appendix 5). The harness-force transducer assembly was adjusted depending on the athlete’s individual sitting height. Maximal isometric trunk flexion and extension tests were performed in randomized sequence. All athletes performed two maximal isometric flexor and extensor tests, each lasting three seconds. For each trial, athletes received a test instruction prior to test performance to act “as forcefully as possible”. Movements of the lower body were strictly controlled. Trials with an identified initial countermovement were discarded (by visual inspection of the force time curve). The force signal was amplified using a charge amplifier (type 5011B; Kistler^®^), analog-to-digital converted (Vicon MX Ultranet Analog Output Receiver, Vicon Motion Systems, California, USA), sampled at 1000 Hz, and finally stored on a computer running Vicon Nexus (Vicon Motion Systems Version 2.16, Vicon Motion Systems Ltd., USA).

#### Local Trunk Muscle Strength Endurance

To assess local TMS endurance, the Bourban test [33] was applied for the ventral, lateral, and dorsal trunk muscles. The ventral Bourban test, affords athletes to be in prone plank position, supported on their elbows and toes. A lower horizontal reference rod was adjusted at the level of the iliac crests, and athletes were instructed to lift their feet al.ternately five centimeters above the ground which was timed by a metronome (two seconds per foot). The test was terminated after the third loss of contact with the reference rod or upon exhaustion. As dependent variable, test time was recorded to the nearest second.

The lateral Bourban test was conducted in side bridge position, with athletes extending their legs, placing the upper foot on top of the lower, and aligning the supporting shoulder above the corresponding elbow. A reference rod was positioned at the level of the superior iliac crest, and athletes continuously raised and lowered their hips in sync following timing of a metronome (two seconds per cycle) while avoiding unloading their body on the mat. The test was terminated after the third warning for either loss of contact with the rod or unloading, and the time until test termination was recorded as dependent variable.

For the dorsal Bourban test, athletes were lying in prone position on a wooden box with the upper border of the iliac crest being at the edge of the wooden box. Arms were crossed in front of the chest, legs extended, and feet secured with padded straps. The horizontal position (0°) was monitored using a mechanical goniometer. Athletes lowered their trunk by 30° which was tested through a mechanical goniometer, while reference rods applied at the level of the thoracic spine and sternal angle were used to control for the trunk’s range of motion during the test. During testing, athletes continuously raised and lowered their trunk in sync with a metronome (two seconds per cycle). The test was terminated in case athletes failed to reach the upper reference rod for the third time, and the time until termination was recorded as dependent variable. Previously, good CVs were reported for the ventral, dorsal and lateral Bourban test, amounting to 14.1%, 11.7%, and 14.6%, respectively [33].

#### Physiological Cycling Economy

Physiological cycling economy was tested using the K5 mobile spiroergometry device (Cosmed, Germany). This device has shown excellent reliability (ICC > 0.991 for V̇O₂ and V̇CO₂, CV = 3.3%) [34]. For all measurements, the mixed chamber method was used and data were averaged over ten seconds [35]. Room temperature and humidity were controlled with a thermos- and hygrometer and were similar during pre and post-tests. Cosmed OMNIA 2.4.2 software was used to record and analyze spiroergometry data. CEO₂ was defined as the rate of oxygen consumption at standardized power output during cycling on the treadmill and it was adjusted for each individual’s body mass. Data were averaged over the entire test protocol.

$${\mathrm{CE}}{{\mathrm{O}}_2}~=\frac{{\dot {V}{O_2}~ \cdot Body~Mass~\left( {kg} \right)}}{{60~ \cdot ~1000}}$$

Cycling economy (CE CO₂) was defined as the rate of CO₂ exhalation at standardized power output adjusted for the body mass of the individual, averaged over the entire test protocol.

$$CEC{O_2}~=\frac{{\dot {V}C{O_2}~ \cdot Body~Mass~\left( {kg} \right)}}{{60~ \cdot ~1000}}$$

The respiratory exchange ratio (RER) was calculated as the ratio of CO₂ to V̇O₂. Data were averaged over the entire test protocol, with values indicating substrate utilization (0.7 for fat oxidation, 1.0 for carbohydrate oxidation) [6].

$$ RER = \frac{{\dot{V}CO_{2} ~}}{{\dot{V}O_{2} ~}} $$

Heart rate (HR) was measured using Polar heart rate chest belts (Polar, Kempele, Finland), worn securely around the chest according to the manufacturer’s guidelines. Data were transmitted wirelessly to a Garmin Edge 540 bicycle computer (Garmin, USA) and recorded in real-time. Average HR values and peak HR were calculated over the entire testing period. As dependent variables for the assessment of physiological cycling economy, we used CEV̇O₂, CEV̇CO₂, RER and HR.

#### Mechanical Factors Affecting Cycling Economy

Mechanical factors affecting CE was tested using the Garmin XC200 Powermeter and the Garmin Edge 540 bicycle computer (Garmin, USA). The dependent variables were cadence, power, balance left/right, torque effectiveness left/right, pedal smoothness left/right and seated time in %. Torque effectiveness reflects the proportion of positive torque applied during the pedal stroke through dividing the net positive power (total positive power minus any negative power) by the total positive power (%). A torque effectiveness of 100% indicates that the applied power contributes to forward motion, with no counterproductive negative torque. The pedal smoothness evaluates how evenly the power is distributed throughout the pedal cycle. It has been computed by dividing the average power by the peak power within a stroke (%). A pedal smoothness of 100% signifies uniform power delivery throughout the entire pedal rotation [36]. For the context of this study, these parameters were summarized under the umbrella term “mechanical CE” (Table 2). To further assess lateral displacement of the bicycle, a self- manufactured wireless accelerometer (construction plan can be viewed in supplementary material, appendix 6) was used. The device was connected to the frame of the bike in a standardized and padded position just behind the stem of the frame (appendix 4). Data were sampled at 1000 Hz and collected via Vicon Nexus software. A second-order Butterworth band-pass filter (0.5–20 Hz) was applied for the accelerometer data. The cut-off frequencies were determined via spectral analysis using fast-fourier transformation, ensuring isolation of relevant displacement signals while minimizing noise and drift. Average displacement per minute over the entire length of the test protocol were calculated by integrating acceleration signals obtained from analogue biomechanical data. The raw signal was rectified and corrected for baseline shifts and scaled to units of ${m/s}^{2}$ using the gravitational constant $(g=9.81\hspace{0.17em}m/{s}^{2}$). To further minimize noise and capture dynamic fluctuations, a sliding window integration technique at $Nw=100$ was applied to compute velocity and displacement. The displacement, representing cumulative sway distance, was computed as the time integral of velocity. As dependent variables for mechanical CE, we used balance left/right, torque effectiveness left/right, pedal smoothness left/right, lateral displacement.

#### Hormonal Analyses

Before testing, athletes were asked to collect saliva by not swallowing with their mouth closed for two minutes to then provide a sample using the passive drool method [24]. Samples were immediately frozen at -20 °C and sent to an external laboratory (Dresden LABservice GmbH, Dresden, Germany) for further analysis. Here, high throughput liquid chromatography–tandem mass spectrometry assay method was used to determine cortisol, progesterone and estrogen levels. This method has been widely used and presents good intra-assay coefficient of variation (cortisol: 5.4%, progesterone: 2.8%, estrogen: 3.0%) [37]. While the coefficient of variation for cortisol slightly exceeds the 5% threshold often suggested for performance-related measures, such values are considered acceptable for salivary hormone analyses using liquid chromatography–tandem mass spectrometry and are in line with previous endocrine research [37].

### Training Protocols

Overall, 24 exercise sessions were conducted over the eight week training period in both the TMS and the active CON group. Total training volume and intensity were similar between groups. After a ten-minute warm-up, consisting of jogging at moderate intensity (~ 60–70% HRmax), the TMS group completed 30 min of global TMS training. The training program focused on exercises primarily targeting the global trunk muscles, including the abdominal muscles (m. abdominalis) and spinal extensors (m. extensor spinae). Athletes performed strength training exercises for the global trunk muscles with external resistance (e.g., dumbbells, barbells, or machines) in three sets of ten repetitions, selecting a load at 75–80% of their one-repetition-maximum (1RM). The 1RM was determined during familiarization sessions using a standardized incremental loading protocol, in which resistance was progressively increased until the maximum load that could be lifted once with proper exercise technique. Adequate rest was provided between attempts, and 1RM testing was supervised by qualified staff to ensure safety and correct execution. Inter-set rest periods of 60–90 s were provided. Load progression was realized on a weekly basis to ensure training progression and continued adaptation. Training loads were progressively adjusted on a weekly basis to ensure adequate overload and continued adaptation. Where applicable, load progression was based on repeated 1RM assessments. For exercises in which direct 1RM testing was not feasible (e.g., pull-up bar windshield wipers), external resistance (e.g., ankle weights or weighted vests) was incrementally added to ensure that athletes reached volitional fatigue within the prescribed repetition range. The complete workout regime can be found in the supplementary material, appendix 7. Training sessions were supervised by qualified coaches to ensure proper exercise execution and adherence. The CON group followed their traditional strength training routine, consisting of 30 min of strength exercises for the lower and upper body without performing specific global or local trunk muscle exercises. Athletes performed exercises in three sets of ten repetitions at a load of 75–80% of the 1RM. The active CON training program was also supervised by qualified coaches.

In addition to strength training, both groups completed similar bike workouts as the athletes (TMS and CON groups) trained the on-bike training together. As the study period was during the German autumn-/ winter time and during athletes’ off-season, on-bike training was irregular due to weather conditions. Athletes completed two to three on-bike training sessions per week, lasting between one and three hours each. Trail biking was performed at self-selected intensity without structured load control. The training hours on the bike were monitored by fitness wearables (e.g., Garmin Fenix watch) used by the participants. Participants were instructed to maintain their usual MTB training practices, including recording data for their personal training documentation. The research team did not intervene in or influence the content or intensity of MTB training.

### Statistical Analyses

Normal data distribution and homoscedasticity were tested and confirmed using the Kolmogorov-Smirnov Test [38] and the Levene test [39]. Independent t-tests were applied to identify possible baseline differences between groups. A mixed ANCOVA (within subject factor time: pre/ post, between subject factor groups: TMS / CON) was performed to determine main group and time effects as well as group-by-time interactions. To further investigate the potential influence of menstrual-cycle related hormonal fluctuations on training effects, hormonal data were used as covariates. In case of significant group-by-time interactions, Bonferroni adjusted post-hoc tests were computed. Effects sizes (η²) were taken from ANCOVA output and transformed into Cohen’s *d* using the following formula: *d* = √(η² * (1 - η²)) * (1 / √2) [40]. Within group Cohen’s *d* were calculated using the following equation: *d* = mean$pre$ - mean$post$/SD$pre$ [41, 42]. Cohen’s *d* can be interpreted as: < 0.5 = small effect, 0.5–0.8 = medium effect and > 0.8 = large effect [43]. The level of statistical significance was set at *p* < 0.05. All analyses were computed using the Statistical Package for Social Sciences (SPSS) version 29 (SPSS inc., Chicago, Illinois, USA).

## Results

### Training Program

All athletes received interventions as allocated. Initially, 25 female MTB athletes met the inclusion criteria. One drop-out occurred over the course of the study due to a non-intervention or test-related MTB training injury (Fig. 1). The final data set comprised 24 athletes with twelve athletes in the TMS group and twelve athletes in the CON group (Table 1). The mean adherence rate to training was 95 ± 5%. There were no significant between-group baseline differences for any of the assessed outcome variables indicating comparable starting conditions across groups (Table 1). No training- or test-related injuries occurred during the study.

### Body Composition

No significant main time or group effects and no significant group-by-time interactions were found for body composition parameters, including fat mass, lean mass, and the BMI (all *p* > 0.05, d = 0.03–0.39).

### Physical Fitness Tests

#### Maximal Isometric Trunk Muscle Strength

Table 3 presents pre and post-intervention values for all measures of maximal isometric flexor and extensor TMS, as assessed in both groups.

**Table 3:** Group-specific mean values and standard deviations for maximal isometric trunk force from pre to post (p-values and effect sizes [Cohen’s d])

	Active control				Trunk muscle strength						
									Main effect of group	Main effect of time	Interaction of group*time
Variable	PRE	POST	Δ %	95% CI	PRE	POST	Δ %	95% CI	(p-value, Cohen’s d)	(p-value, Cohen’s d)	(p-value, Cohen’s d)
**Ventral**	46.0 ± 7.0	47.5 ± 5.80	3.3%	44.55–50.45	42.27 ± 11.38	62.33 ± 10.64	47.5%	35.04–49.50	0.25 (0.26)	< 0.001 (1.28)	< 0.001 (1.10)
[kg]											
**Dorsal**	74.25 ± 22.72	69.25 ± 16.63	-7%	59.82–88.69	62.92 ± 24.41	87.92 ± 29.34	39.7%	47.42–78.43	0.69 (0.08)	0.001 (0.78)	< 0.001 (01.17)
[kg]											

kg = Kilograms

**Fig. 2:**
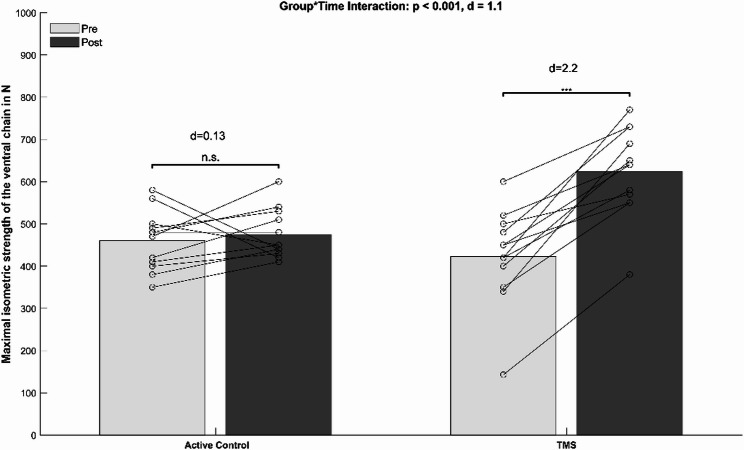
Mean values (bars) and individual data (lines) for the ventral maximal isometric trunk muscle test. Legend: d, Cohen’s d; * = *p* < 0.05; ** = *p* < 0.005. Abbreviations: TMS = Trunk muscle strength training group; N = Newton; n.s. = not significant

**Fig. 3:**
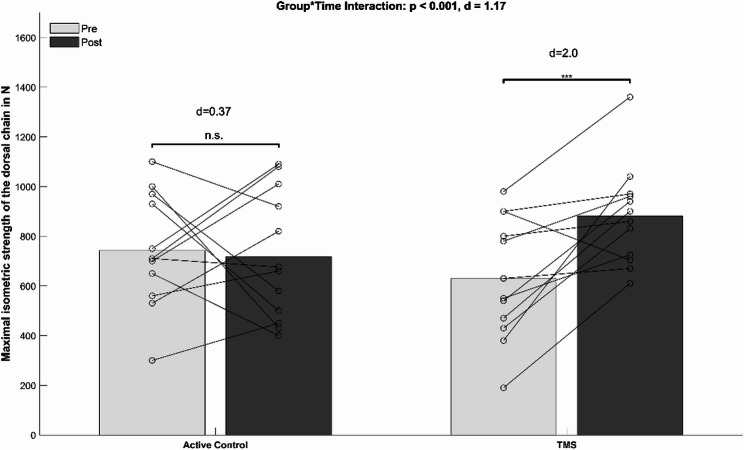
Mean values (bars) and individual data (lines) for the dorsal maximal isometric trunk muscle test. Legend: d, Cohen’s d; * = *p* < 0.05; ** = *p* < 0.005. TMS = Trunk muscle strength training group; N = Newton; n.s. = not significant

The ANCOVA analysis (Table 3) revealed a significant group-by-time interaction for the trunk flexors (*p* < 0.001, d = 1.10). Post-hoc tests indicated a significantly enhanced maximal trunk flexor force in the TMS group (*p* < 0.001, d = 2.2, Δ + 47.5%) and no significant performance change in the CON group (*p* = 0.55, d = 0.19, Δ + 3.3%) (Fig. 2). Significant group-by-time interaction was also found for trunk extensors (*p* < 0.001, d = 1.17). Post-hoc tests indicated a significant improvement for the TMS group (*p* < 0.001, d = 2.0, Δ + 39.7%) but not the CON group (*p* = 0.23, d = 0.37, Δ-7%) (Fig. 3).

#### Trunk Muscle Strength Endurance

**Fig. 4:**
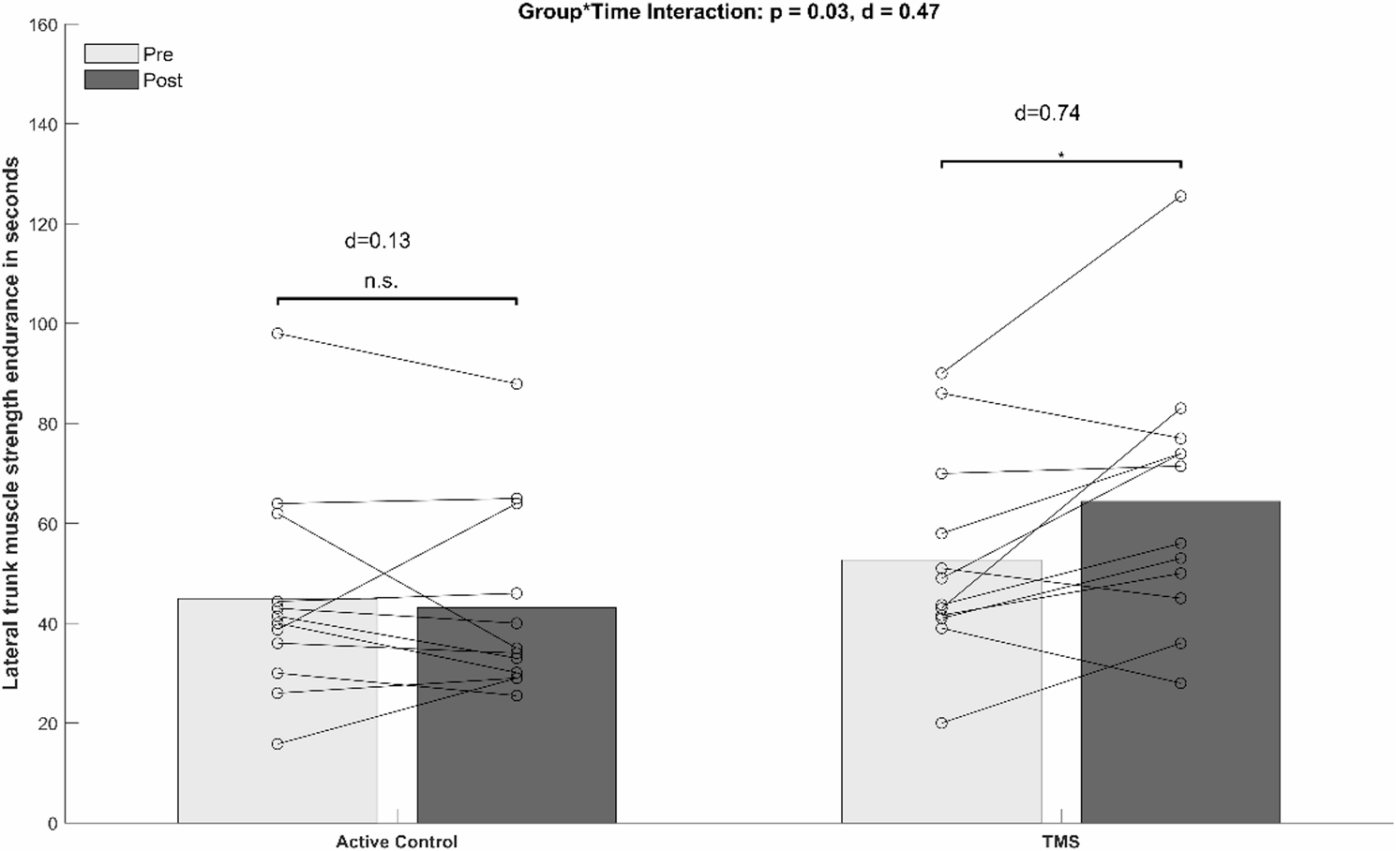
Mean values (bars) and individual data (lines) for the lateral trunk muscle endurance test. Legend: d, Cohen’s d; * = *p* < 0.05; ** = *p* < 0.005. TMS = Trunk muscle strength training group; n.s. = not significant

Table 4 presents pre and post data for all measures of trunk muscle strength endurance. The ANCOVA analysis (Table 4, 5) showed significant group-by-time interactions for the lateral trunk muscles (*p* = 0.03, d = 0.47). Post-hoc tests indicated a significantly improved lateral trunk muscle strength for the TMS group (*p* < 0.03, d = 0.74, Δ + 22.2%) but not for the CON group (*p* = 0.65, d = 0.13, Δ-3.9%) (see Fig. 4).

**Table 4:** Group-specific mean values and standard deviations for trunk muscle endurance from pre to post (p-values and effect sizes [Cohen’s d])

	Active control				Trunk muscle strength						
									Main group effect	Main time effect	Group-by-time interaction
Variable	PRE	POST	Δ %	95% CI	PRE	POST	Δ %	95% CI	(p-value, Cohen’s d)	(p-value, Cohen’s d)	(p-value, Cohen’s d)
Ventral	129.20 ± 60.56	116.25 ± 90.59	-10.3%	90.72-167.68	146.11 ± 62.54	173.20 ± 107.05	18.5%	105.18–221.3	0.25 (0.26)	0.57 (0.11)	0.12 (0.33)
[s]											
Dorsal	86.11 ± 24.89	71.8 ± 25.82	-16.6%	70.30-101.92	91.01 ± 25.24	88.07 ± 20.21	-3.3%	75.23-100.91	0.19 (0.28)	0.16 (0.31)	0.34 (0.2)
[s]											
Lateral	44.94 ± 21.44	43.2 ± 19.2	-3.9%	31.32–58.56	52.69 ± 20.3	64.41 ± 25.91	22.2%	47.95–80.87	0.09 (0.35)	0.11 (0.35)	0.03 (0.47)
[s]											

s = Seconds

**Table 5:** Group-specific mean values and standard deviations for mechanical CE from pre to post (p-values and effect sizes [Cohen’s d])

	Active control				Trunk muscle strength						
									Main group effect	Main time effect	Group-by-time interaction
Variable	PRE	POST	Δ %	95% CI	PRE	POST	Δ %	95% CI	(p-value, Cohen’s d)	(p-value, Cohen’s d)	(p-value, Cohen’s d)
Lateral displacement	6.39 ± 1.34	7.16 ± 1.37	12%	6.29–8.03	6.48 ± 1.08	5.21 ± 1.37	-19.6%	4.34–6.08	0.059 (0.43)	0.37 (0.2)	0.001 (0.8)
[m·min⁻¹]											
Avg Cadence	78.55 ± 4.8	76.18 ± 7.45	-3.1%	71.45–80.91	82.92 ± 6.30	80.83 ± 6.47	-2.5%	76.72–84.94	0.08 (0.4)	0.04 (0.48)	0.89 (0.032)
[min⁻¹]											
Balance L/R	50.18 ± 2.78	50.09 ± 2.3	-0.18%	48.63–51.55	49.00 ± 1.18	47.82 ± 3.31.34	-2.4%	45.72–49.92	0.073 (0.42)	0.27 (0.25)	0.25 (0.21)
[%]											
Torque Effectiveness	72.4 ± 6.34	71.8 ± 6.52	-0.8%	67.66–75.94	69.64 ± 4.67	67.45 ± 8.53	-3.2%	63.03–72.87	0.11 (0.38)	0.49 (0,16)	0.69 (0.09)
[%]											
Pedal Smootheness L/R	22.10 ± 2.28	21.80 ± 2.39	-1.4%	20.28–23.32	21.09 ± 2.11	20.45 ± 3.32	-3.1%	18.34–22.56	0.2 (0.3)	0.57 (0.15)	0.8 (0.05)
[%]											
Avg Power·kg⁻¹	2.29 ± 0.81	2.22 ± 0.76	-3.2%	1.74–2.7	2.43 ± 0.33	2.23 ± 0.73	-8%	1.77–2.69	0.78 (0.05)	0.27 (0.23)	0.59 (0.12)
[Watt]											
Seated time [%]	98.7 ± 2.4	99.1 ± 1.7	0.4%	98.1–100	97.9 ± 4.4	98.8 ± 2.4	1%	96.5–100	0.43 (0.33)	0.36 (0.28)	0.57 (0.18)

###  Physiological Cycling Economy

Table 6 presents pre and post data for all measures of physiological CE.

The ANCOVA analysis (Table 6) revealed significant main time effects for V̇O₂ and V̇CO₂ (*p* = 0.001, d = 0.77; *p* = 0.01, d = 0.4, respectively). However, the analysis failed to indicate significant group-by-time interactions (supplementary material, appendix 8, Fig. 6).

### Mechanical Cycling Economy

Table 5 presents pre and post data for all measures of trunk mechanical CE.

**Table 6:** Group-specific mean values and standard deviations for physiological CE from pre to post (p-values and effect sizes [Cohen’s d])

	Active control				Trunk muscle strength						
									Main groups effect	Main time effect	Group-by-time interaction
Variable	PRE	POST	Δ %	95% CI	PRE	POST	Δ %	95% CI	(p-value, Cohen’s d)	(p-value, Cohen’s d)	(p-value, Cohen’s d)
Average heart rate	162.00 ± 14.14	159.92 ± 15.52	-1.3%	150.06 -169.78	161.67 ± 10.84	159.25 ± 12.54	-1.52%	151.28 -167.22	0.92 (0.01)	0.08 (0.38	0.89 (0.03)
[beats·min⁻¹]											
Max heart rate	187.25 ± 11.22	186 ± 12.51	-1%	178.05 -193.95	185.83 ± 10.08	185.5 ± 9.92	-0.1%	179.2 -191.8	0.82 (0.04)	0.46 (0.15)	0.67 (0.09)
[beats·min⁻¹]											
V̇O₂	36.89 ± 4.65	35.56 ± 4.82	-3.7%	32.5–38.62	36.6 ± 4.02	33.49 ± 3.03	-8.5%	31.56–35.42	0.46 (0.15)	0.001 (0.77)	0.16 (0.39)
[ml·kg⁻¹·min⁻¹]											
V̇CO₂	35.06 ± 4.09	34.6 ± 4.74	-1.3%	31.59–37.61	34.82 ± 4.43	31.56 ± 2.0	-9.6%	30.29–32.83	0.79 (0.17)	0.01 (0.40)	0.08 (0.27)
[ml·kg⁻¹·min⁻¹]											
RER	0.96 ± 0.05	0.977 ± 0.06	-1.74%	0.94–1.02	0.95 ± 0.05	0.94 ± 0.06	-1.1%	0.9–0.98	0.32 (0.21)	0.82 (0.04)	0.2 (0.26)
[-]											

Max heart rate = maximal heart rate; V̇O₂ = oxygen uptake; V̇CO₂ = carbon dioxide output; RER = respiratory exchange ratio

**Fig. 5:**
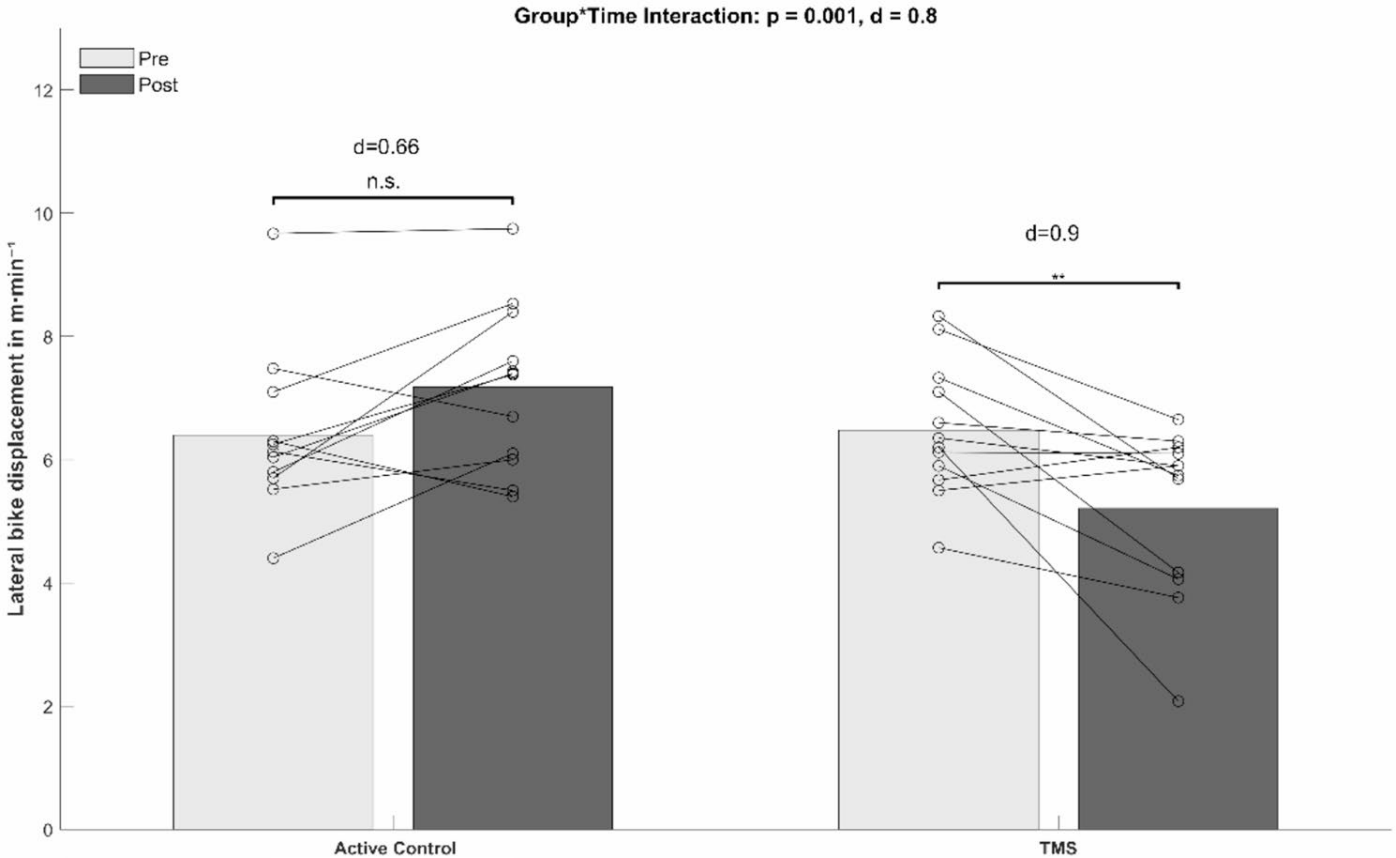
Mean values (bars) and individual data (lines) for lateral bike displacement. Legend: d, Cohen’s d; * = *p* < 0.05; ** = *p* < 0.005. TMS = Trunk muscle strength training group; n.s. = not significant

The ANCOVA analysis (Table 3) revealed significant group-by-time interactions for the parameter lateral bike displacement (*p* = 0.001, d = 0.8). Post-hoc tests indicated a significantly reduced lateral displacement for the TMS group (*p* = 0.01, d = 0.9, Δ-19.6%) and no significant changes for the CON group (*p* = 0.06, d = 0.66, Δ + 12%) (Fig. 5). In addition, lateral bike displacement was analyzed separately for the climbing sections of the treadmill protocol. The ANCOVA revealed a significant group-by-time interaction for lateral displacement during climbing (*p* = 0.003, d = 0.74) (supplementary material, appendix 8, Fig. 8). Post-hoc analyses showed a significant reduction in lateral displacement in the TMS group (Δ − 14.7%), whereas lateral displacement increased in the CON group (Δ + 14.2%). No significant main effects of time or group were observed (supplementary material, appendix 8, Table 7). No significant main time and group effects, and no significant group-by-time interactions were found for the remaining mechanical CE parameters.

### Hormones

The ANOVA analysis revealed no significant main effects of time, group, and group-by-time interactions for cortisol, progesterone and estrogen levels or the P/E ratio (progesterone divided by estrogen) over the course of the training period.

No significant moderating effects on the dependent variables were observed in the ANCOVA analyses with any of the hormonal covariates (*p* > 0.05, d = 0.09–0.2).

## Discussion

In this randomized controlled trial, we examined the effects of TMS training on TMS, CE and mechanical factors affecting CE in trained and highly-trained (Tier 2–3) female MTB athletes. The study was conducted during the off-season over a training period of eight weeks. To our knowledge, this is the first study to use a fully standardized instrumented race course simulation on a 5 × 3 m treadmill and implementing global TMS training in trained and highly-trained female MTB athletes. The main findings indicated significant improvements in the TMS group for maximal isometric TMS in the dorsal and ventral chain, lateral TMS endurance and lateral bike displacement. However, no significant changes were found for physiological CE (V̇O₂ and V̇CO₂) or body composition. Further, the analyzed hormonal covariates did not moderate the observed effects.

The improvements in maximal isometric ventral and dorsal TMS align with previous research conducted in athletic populations. For instance, Zinke and colleagues [20] found significant and medium effects on peak torque of the trunk rotators at different angular velocities following an eight week isokinetic TMS training. No training-induced gains were found for TMS endurance in male world-class (Tier 5) canoe sprinters aged 25.6 ± 3.3 years.

This study revealed medium effects of TMS training on lateral TMS endurance. The difference in findings between our study and that of Zinke et al. might be due to their use of single-mode and monotonous strength training using an isokinetic dynamometer, enabling greater focus on the global trunk musculature. We had larger exercise variability included in our TMS training program ranging from machine-based to free weight trunk muscle exercises. Similar to our study results, Zinke et al. [20] did not detect any significant changes in body mass or composition.

When examining male and female cyclists aged 34.1 ± 9 years (Tier 2), Wieseman et al. [44] found positive medium-sized adaptations for maximal TMS following a four-week TMS training. Similar to our results, body composition remained unchanged. These findings indicate that the intervention did not impact on body composition, suggesting that the observed performance changes are not due to changes in fat or lean mass. In an effort to summarize the available literature on TMS training, Saeterbakken et al. [10] aggregated findings from 31 studies with male and female athletes (Tier 1–3) from different sports (soccer, swimming, running, golf) aged 11–37 years in the form of a meta-analysis. The results showed significant small-to-large effects of TMS training on TMS, physical fitness, and sport-specific performance when compared to active controls. Interestingly, athletes’ age was found to significantly moderate agility outcomes with larger TMS training effects in youth [10]. Further supporting these results, a meta-analysis summarizing nine studies with a total of 228 male and female (Tier 1–3) individuals was conducted by Prieske et al. [45]. These authors showed a large TMS training effect on measures of TMS. Hence, based on the results of our study and findings from the literature [10, 44, 45], TMS training seems to present a well-suited exercise regime to improve maximal TMS in athletes in general and in female MTB athletes in particular.

Various studies have explored the effects of general strength training and TMS training on CE [5, 44, 46–48]. For example, Vikmoen and colleagues [47] compared cycling performance adaptations following strength training between males and females and concluded that strength training positively impacted cycling performance. Yet, the results conducted with males only cannot be used and extrapolated to female populations. Therefore, results from studies including female athletes should be prioritized when comparing our study findings with those of the existing body of literature. Wiseman et al. [44] evaluated the effects of a four-week TMS training on cycling performance in trained (Tier 2) male (34) and female (6) cyclists aged 34.1 ± 9 years. In this study, the TMS group showed positive changes in CE, cycling speed, power and average heart rate.

In another study, Park et al. [49] analyzed isokinetic TMS in relation to pedaling power in 200 male cycling athletes aged 24.5–26.1 years with Tier 2–3 training and performance calibre. Results from these authors showed that cyclists with higher pedaling power had greater isokinetic trunk extension and flexion strength [49]. In another study, Geißler et al. [48] examined potential associations between TMS measured under isokinetic conditions and endurance performance in a large cohort of 1,149 individuals, predominantly male soldiers (1,083 male, 66 female) with a mean age of 38.3 ± 12.9 years. For this purpose, the authors retrospectively analyzed 1,334 bicycle ergometry tests and correlated these analyses with TMS data. The authors reported a moderate correlation between maximal cycling power and TMS (*r* = 0.312–0.398) [48]. These findings indicate an association between TMS and cycling-related power output. However, as both studies are cross-sectional in nature, they do not provide evidence for causal or training-induced effects. Rather, the observed relations likely reflect the well-established link between absolute strength and absolute power.

Sitko et al. [50] examined the effects of TMS training versus traditional strength training and control on power output in 36 trained (Tier 2) male road cyclists aged 28.8 ± 4.2 years. The twelve-week training program was conducted during the off-season, and power outputs were assessed pre and post training over 5 s, 60 s, 5 min and 20 min. The authors observed significant performance improvements in the TMS group. However, the traditional strength training group demonstrated even greater gains compared to the control group. These contrasting results might be due to differences in TMS training methodology. While our TMS protocol specifically targeted the global trunk musculature, Sitko et al. [50] used exercises such as planks or prone back extensions, targeting mainly the local trunk stabilizers. They further conducted tests using a fixed Tacx system, which eliminated the need for bicycle balancing. This test set-up created an additional deviation compared to our treadmill-based mountain biking setup, where dynamic stability plays a crucial role. The reported discrepancy in findings underscores the importance of tailoring TMS protocols to the specific demands of different cycling disciplines, such as mountain biking and road cycling. However, it should also be acknowledged that the traditional strength training performed in the active control group of the present study is comparable to the conventional strength training program employed by Sitko et al. [50]. As performance outcomes and maximal power were not assessed in the present study, direct comparisons of training-induced performance changes between studies should be interpreted with caution.

While the above-described studies suggest a possible impact of TMS training on CE and cycling performance, they do not explain the underlying physiological mechanisms driving these improvements. In an attempt to contribute towards closing this research gap, we further examined adaptations in mechanical CE following TMS training. The metabolic cost of maintaining balance during cycling has been studied by Miller et al. [15] and McDaniel et al. [16], both concluding that postural balance is a significant contributor to energy expenditure. Similarly, Wilkinson and Kram [51] found that the lean angle of the bicycle influences the rider’s power output. Building on this finding, our study examined athletes’ lateral displacement while cycling on the treadmill in an attempt to quantify energy loss due to inefficient energy transfer throughout the body segments. In our study, we observed a large-size-effect with a significant reduction of lateral bicycle displacement in the TMS group but not in the control group. It is important to mention that this was not the result of altered seated/standing time, as this parameter remained stable in both groups (Table 5). The reduction in lateral bike displacement in the TMS group suggests improved cycling stability, which could translate to better performance. While the exact mechanisms driving physiological CE improvements through TMS training as documented by the aforementioned study [44] remain unclear, we hypothesize that enhanced TMS facilitates more efficient energy transfer between the upper and lower extremities, e.g. by reducing lateral displacement. With reference to the literature, reduced lateral displacement on the bike seems to represent a better mechanical CE and may contribute to physiological CE [1, 17, 51]. Krohm et al. [1] established the first athlete needs analysis for MTB performance. These authors specifically emphasized the role of trunk muscles in providing distal mobility of the limbs through proximal stability of the trunk, which supports effective force transfer through the body segments [20]. In this context, our findings suggest a connection between TMS and mechanical and, although not seen in our study, potentially physiological CE. By reducing lateral bicycle movement, energy transfer efficiency may improve (mechanical CE), reducing metabolic costs and thereby enhancing physiological CE. Furthermore, the observed trend towards significance for V̇CO₂ with a larger reduction in the TMS group compared with the control group (supplementary material, appendix 8, Fig. 6) in this study leads us to hypothesize that TMS predominantly induced anaerobic adaptations in the trunk muscles, resulting in a more efficient anaerobic metabolism indicated by reduced CO₂ exhalation during the cycling tests [6]. This is also in line with the aforementioned results of Sitko et al. [50], where the largest performance gains through TMS training were found in the shortest test time of 5 s, rather than in the longer test intervals (60 s, 5 min and 20 min). In order to investigate this further, we additionally calculated the lateral displacement for the steepest climbing segment of the treadmill protocol in an attempt to isolate the segment with the highest anaerobic demands. Figure 8; Table 7 in supplementary material (appendix 8) show that the effects from the computed segmental analysis point in the same direction as the analysis of the entire protocol. These results suggest that TMS gains are not limited to anaerobic efforts, but affect lateral displacements during all cycling phases.

Additionally, averaging over the entire MTB race protocol may have attenuated the training effects of the outcomes, as the expected effects of TMS training are most likely to occur during the steepest incline sections of the protocol. If the goal is to enable segmental race course analysis in future studies, it is recommended to use breath-by-breath spiroergometry instead of a mixed chamber apparatus.

In our study, hormonal cortisol, progesterone, and estrogen concentrations did not moderate the observed TMS training effects. While this finding suggests that these hormones may not directly influence the outcomes of TMS training or CE in the context of our specific study design, it highlights the complexity of hormonal measurements in trained and highly-trained female athletes. Although hormonal fluctuations did not moderate the observed effects, the relatively small sample size and the presence of menstrual cycle irregularities in some athletes may have limited the statistical power of the study to detect potential hormonal influences. In this context, it is important to point out that five out of 24 enrolled athletes did not have a regular menstrual cycle, possibly altering hormonal fluctuations. This degree of freedom presents a major challenge to produce statistically significant results in a rather small cohort of athletes. Nevertheless, a growing body of the literature emphasizes the importance of considering the menstrual cycle phase in training planning and performance optimization as well as injury prevention [25, 27]. Evidence also increasingly indicates that simple cycle phase questionnaires lack the precision required to reliably predict hormonal fluctuations and, consequently, cannot serve as a valid alternative to direct hormonal testing [52]. These findings underscore the need for future research exploring how hormonal fluctuations across the menstrual cycle may interact with specific training modalities or performance outcomes in populations prone to period irregularities induced by high-training volumes. Specifically designed studies with a larger sample size and over longer training periods are needed to successfully investigate these research questions.

### Study Limitations and Future Directions

This study is not without methodological limitations that should be acknowledged. First, we investigated MTB athletes in a laboratory setting on even surface. However, MTB performance is strongly influenced by highly variable surface conditions. For better external validity, treadmills featuring varying ground conditions (by usage of implemented pertubations etc.) could deliver more realistic experimental conditions. As we solely included athletes of the highest regional performance and training caliber (Tier 2–3), our recruitment pool was limited. To meet our recruitment criteria, we had to widen the age range to 14–22 years of the included female athletes, which resulted in a rather heterogenous study population. We accounted for this problem by matching the athletes according to their age and respective regional/national squad level. The baseline tests have indicated no statistically significant baseline differences between the TMS and the control group, but the limitation remains. In terms of the potentially moderating influence of the hormones, a more homogenic population (exclusion criterion of irregular menstrual cycle) should be implemented in future studies.

Furthermore, the study did not include a passive or non-training control group. Instead, the active control group performed conventional strength training. While this approach was chosen to represent current training practice, it limits the ability to compare the intervention against a true no-training control condition. Since the treadmill protocol predetermined the power output for the post-tests, it was not possible to assess improvements in cycling performance variables such as maximal or average power. This limitation was recognized during the study design, yet the chosen approach was maintained to ensure the measurement of cycling economy under standardized conditions.

Future research should use techniques such as electromyography and 3D- motion capturing in order to investigate the underlying mechanisms through which TMS reduces bike displacement and its potential link to cycling economy. In order to better understand the true effects in the niche of female MTB athletes, it is crucial to aggregate data from this and similar (future) studies.

## Conclusions

TMS training appears a safe training modality that has the potential to improve isometric maximal TMS, lateral TMS endurance and reduce lateral bike displacement in female trained and highly-trained national level (Tier 2–3) MTB athletes. Our data suggest that off-season global TMS training can effectively enhance mechanical CE in female MTB athletes. However, despite the observed reductions in lateral bike displacement, no corresponding improvements in physiological CE were detected in the present study. Findings from this study revealed that menstrual cycle related hormonal fluctuations did not significantly impact on the outcomes of this study. Further research is needed to investigate the mechanisms underlying the observed improvements in mechanical and physiological CE through TMS training in female MTB athletes.

## Supplementary Information

Below is the link to the electronic supplementary material.

Supplementary Material 1.

## Data Availability

The datasets generated and analyzed during the current study are not publicly available due to athletes’ data protection policies but are available from the corresponding author on reasonable request.
