# Supplementary material for: Effects of Core Strength Training on Maximal Trunk Muscle Strength and Cycling Economy in Female Mountain Bikers
Source: Sports Med Open. 2026 Mar 9;12:26. doi: 10.1186/s40798-026-00996-0 (PMC12972400; doi:10.1186/s40798-026-00996-0)
Supplement: Supplementary file 1 — Supplementary Material 1. [file 40798_2026_996_MOESM1_ESM.docx]

**Supplementary Material**

**Journal:** *Sports Medicine – Open*

**Article title:** *Effects of Core Strength Training on Maximal Trunk Muscle Strength and Cycling Economy in Female Mountain Bikers*

**Authors:**
Roland Blechschmied¹, Jana Strahler², Urs Granacher¹*

**Affiliations:**
¹ Department of Sport and Sport Science, Exercise and Human Movement Science, University of Freiburg, Freiburg, Germany
² Department of Sport and Sport Science, Sport Psychology, University of Freiburg, Freiburg, Germany

**Corresponding author:**
Prof. Urs Granacher, PhD
Department of Sport and Sport Science
Exercise and Human Movement Science
University of Freiburg
Sandfangweg 4
79102 Freiburg, Germany
Email: urs.granacher@sport.uni-freiburg.de

**Appendix 1: Questionnaire**

Liebe Teilnehmerin,

im Rahmen unserer Studie möchten wir einige personenbezogenen Faktoren als potentielle Einflussgrößen auf unsere Zielvariablen untersuchen. Dafür bitten wir dich um die Angabe der untenstehenden Daten, die unter anderem deinen Zyklusstatus sowie deinen Lebensstil und deine MTB-Erfahrung umfassen. Solltest du dir bei einer Frage nicht sicher sein, sprich uns gerne an.

Vielen Dank!

1. **Generelle Informationen**

- Name: _________________________
- Geburtsdatum: __________________
- Größe (cm): _____________________
- Gewicht (kg): ____________________

**2. Trainingsphase**

**- Welche Trainingsphase trifft auf Sie zu? (Bitte ankreuzen)**

**[ ] Aufbau**

**[ ] Vorwettkampf**

**[ ] Wettkampf**

**[ ] Reha**

**3. Normaler Trainingsumfang**

**- Durchschnittliche Trainingsstunden pro Woche: ________**

**- Durchschnittliche Trainings-Einheiten pro Woche: ________**

**- Zeitpunkt ihres letzten Trainings vor dem Test (Stunden) _____**

**- Anstrengung des letzten Trainings vor dem Test (1 leicht - 10 maximal ) ______**

**4. Ernährung**

**- Zeitpunkt Ihrer letzten Mahlzeit vor dem Test (Stunden): ________**

**- Konsum von Koffein vor dem Test: (Bitte ankreuzen)**

[ ] Ja

[ ] Nein

**- Verwendung von Nahrungsergänzungsmitteln: (Bitte ankreuzen)**

[ ] Ja

[ ] Nein

**- Wenn ja, welche?________________________________**

**5. Gesundheit:**

**Bitte beantworten Sie die folgenden Fragen mit Ja oder Nein.**

**- Hat Ihnen jemals ein Arzt gesagt, Sie hätten «etwas am Herzen» und Ihnen nur unter medizinischer Kontrolle Bewegung und Sport empfohlen?**

[ ] Ja

[ ] Nein

**- Hatten Sie im letzten Monat Schmerzen in der Brust in Ruhe oder bei körperlicher Belastung?**

[ ] Ja

[ ] Nein

**- Haben Sie Probleme mit der Atmung in Ruhe oder bei körperlicher Belastung?**

[ ] Ja

[ ] Nein

**- Sind Sie jemals wegen Schwindel gestürzt oder haben Sie schon jemals das Bewusstsein verloren?**

[ ] Ja

[ ] Nein

**- Haben Sie Knochen- oder Gelenkprobleme, die sich unter körperlicher Belastung verschlechtern könnten?**

[ ] Ja

[ ] Nein

**- Hat Ihnen jemals ein Arzt ein Medikament gegen hohen Blutdruck oder wegen eines Herzproblems oder Atemproblems verschrieben?**

[ ] Ja

[ ] Nein

**- Kennen Sie irgendeinen weiteren Grund, warum Sie heute keinen maximalen Leistungstest machen sollten?**

[ ] Ja

[ ] Nein

**- Wurde in den letzten 1–2 Jahren ein EKG durchgeführt und war das Ergebnis unauffällig?**

[ ] Ja

[ ] Nein

**6. Verletzungen (letzte 6 Monate): __________________________**

**7. Beschwerden am Testtag: _________________________________**

**8. Allgemeine Befindlichkeit (1-10): ________**

**9. Testmotivation (1-10): ________**

**10. Menstruation:**

**- Mein Menstruationszyklus ist (Bitte ankreuzen):**

**[ ] Regelmäßig**

**[ ] Unregelmäßig**

**[ ] Nicht vorhanden**

**- Aktuell befinde ich mich in folgender Phase meines Zyklus:**

**[ ] Luteal**

**[ ] Menstruation**

**[ ] Ovulation**

**[ ] Follikelphase**

- - **Mein Zyklus dauert normalerweise (Tage): ________**
  - **Meine Zyklusbeschwerden beginnen normalerweise (Tage) vor der Menstruation und enden (Tage) nach der Menstruation: __________ - __________**
  - **Aktuell verhüte ich durch (Methode): ______________________**

**Appendix 2:** Treadmill protocol example


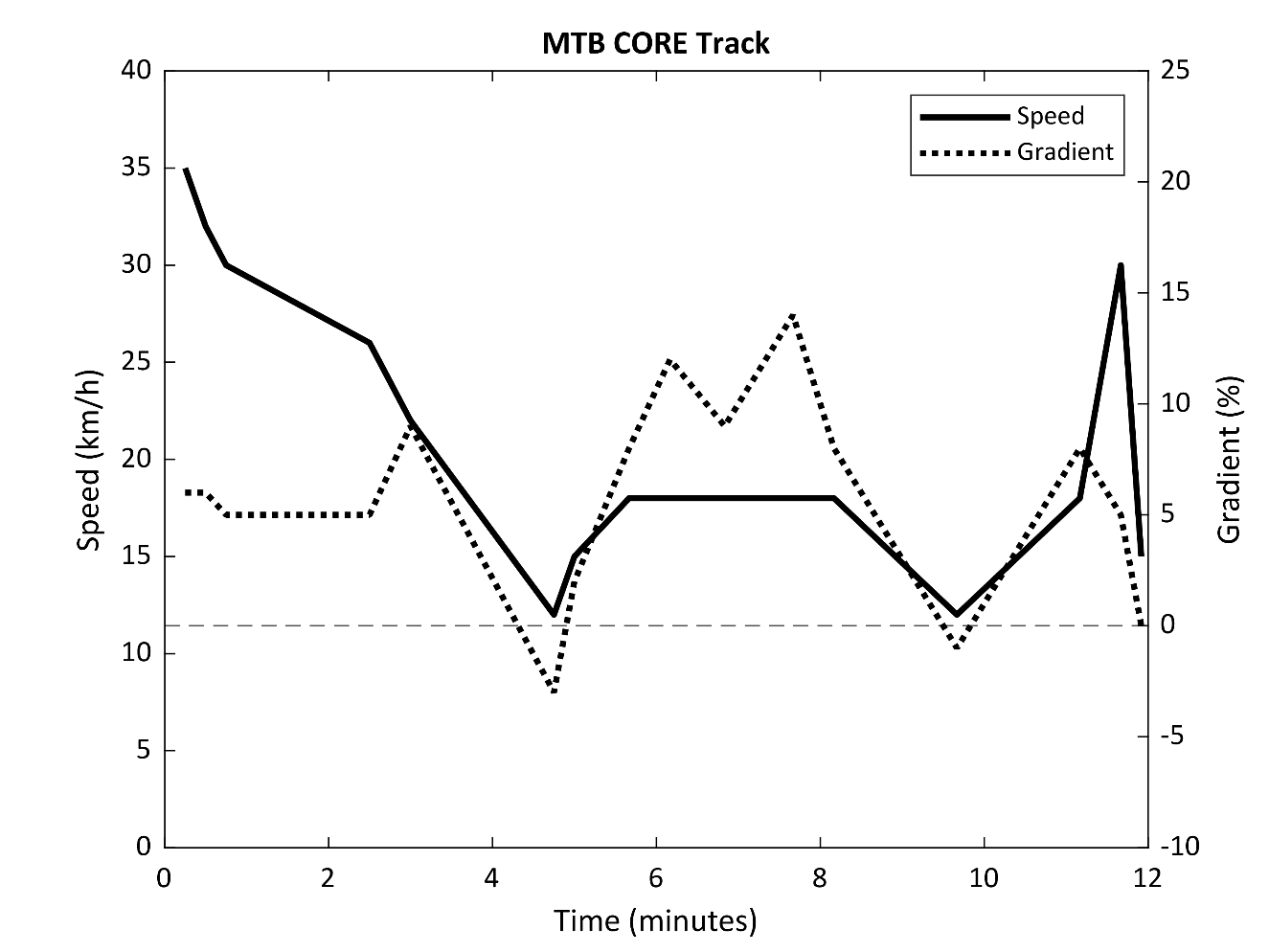


**Appendix 3: Treadmill speed adjustment calculations**


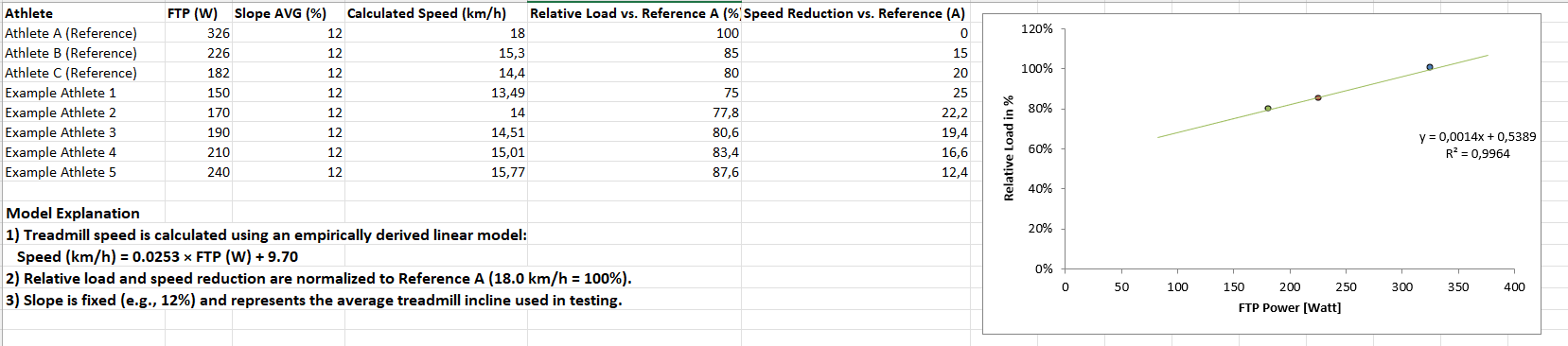


Abbreviations: AVG = Average; FTP = Functional threshold power


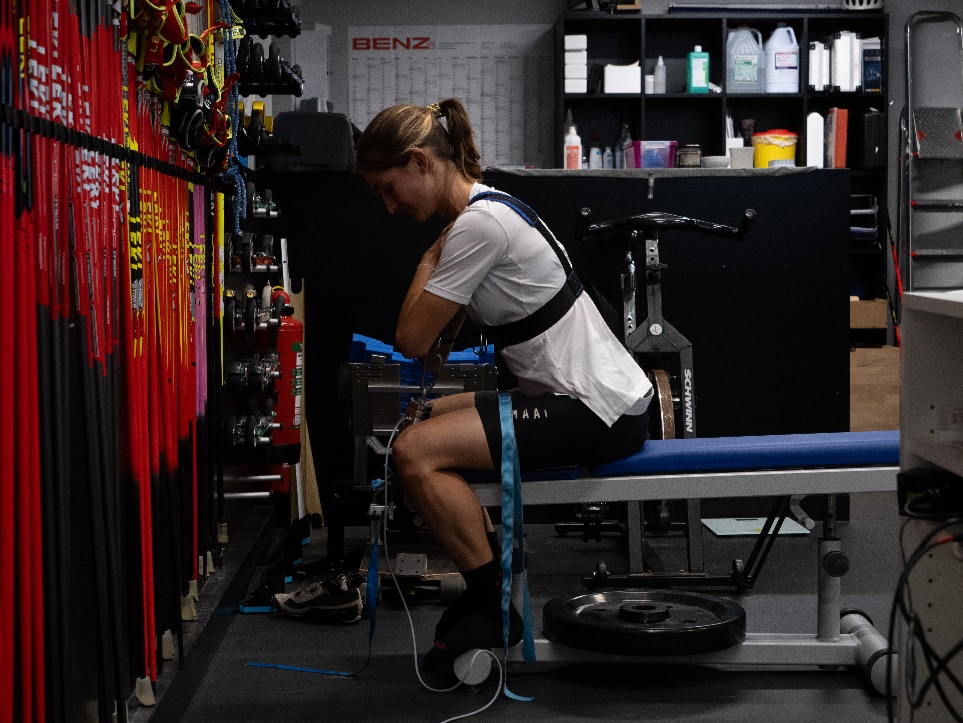


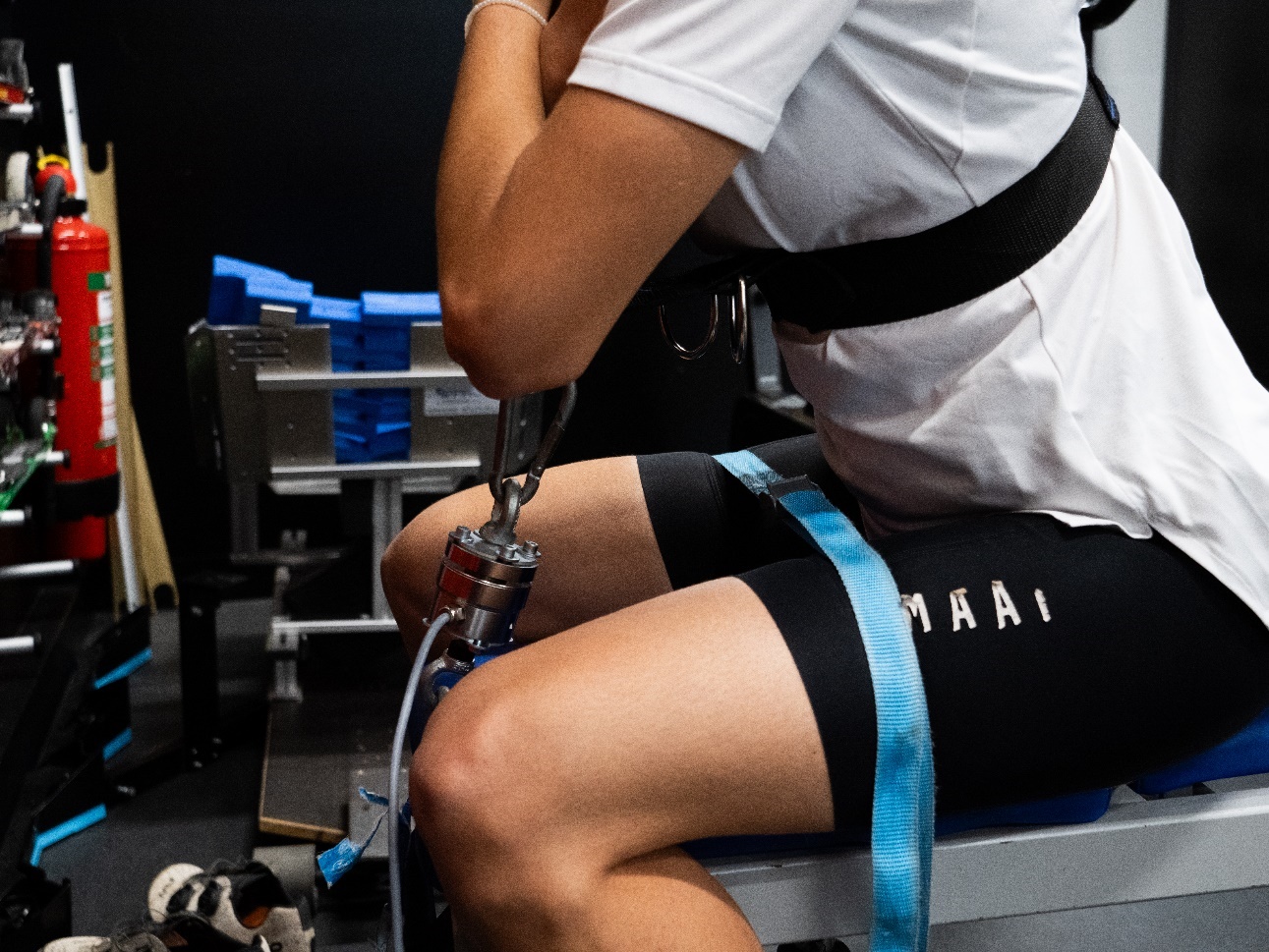
**Appendix 4:** Images of isometric trunk muscle strength testing.

**
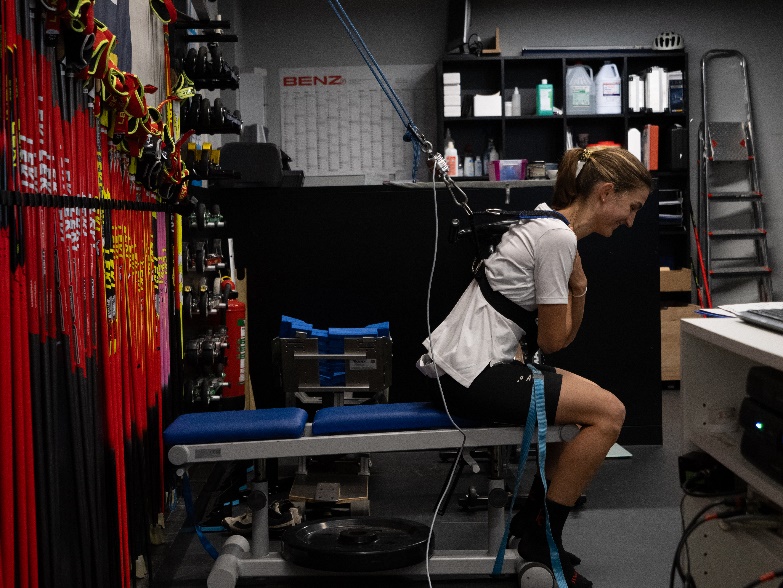
**

**Appendix 5:** Images of the laboratory setup. Written image usage permission was obtained by all displayed persons.
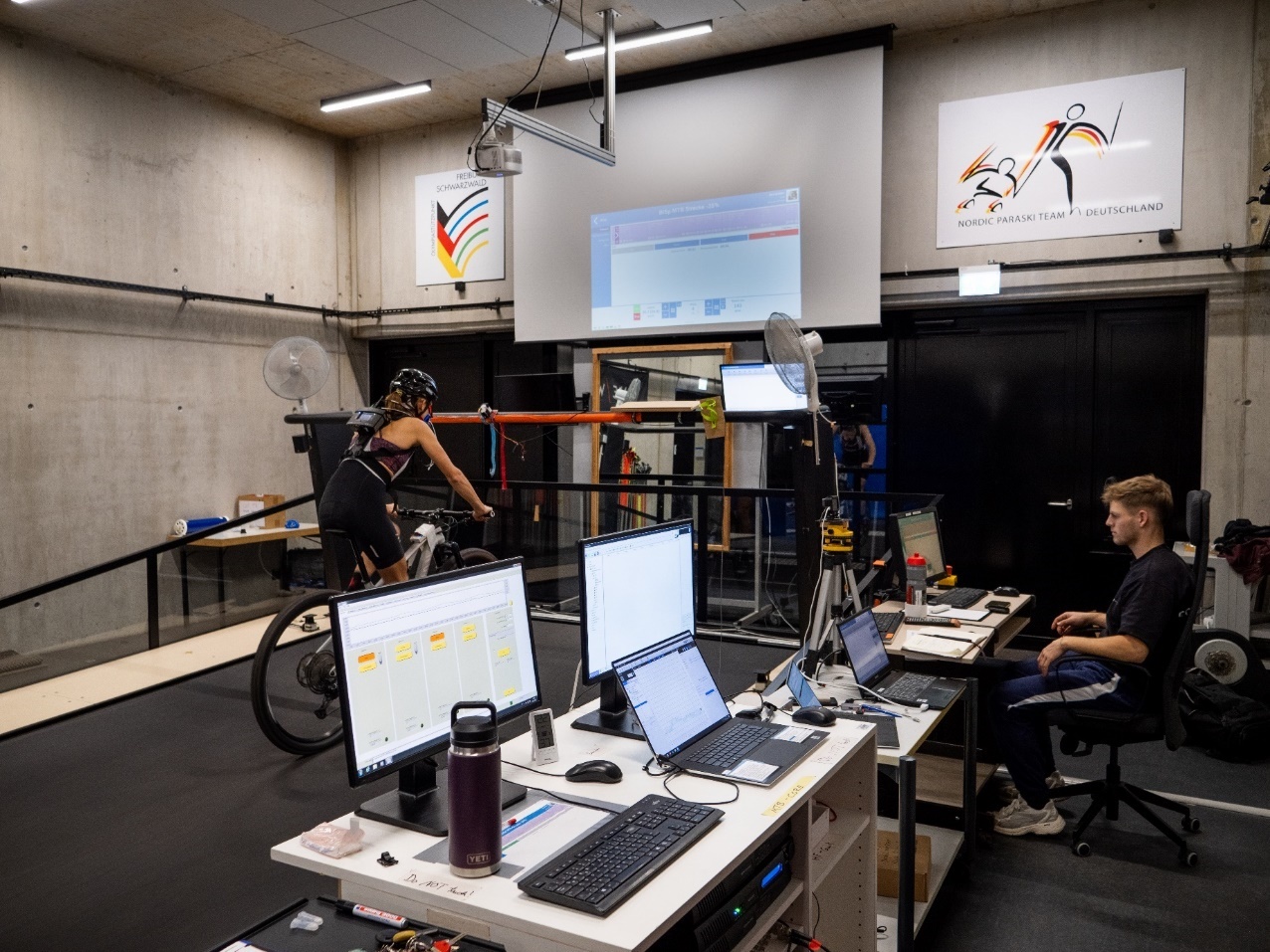

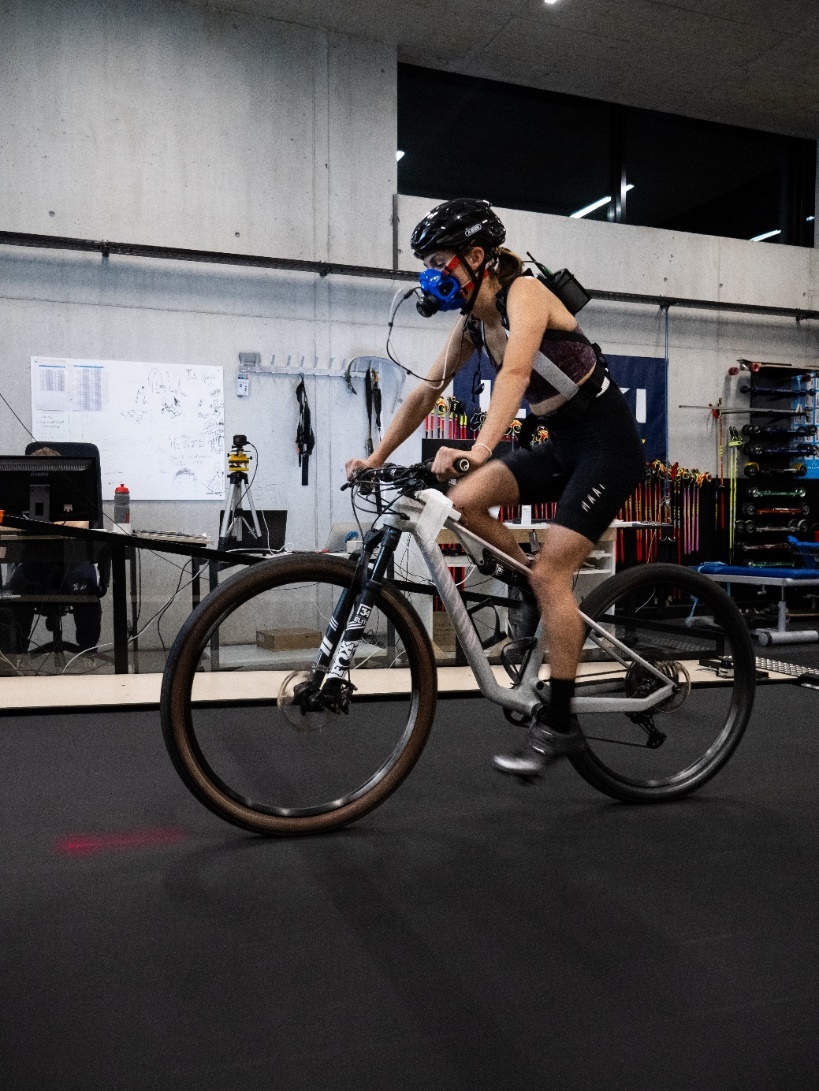


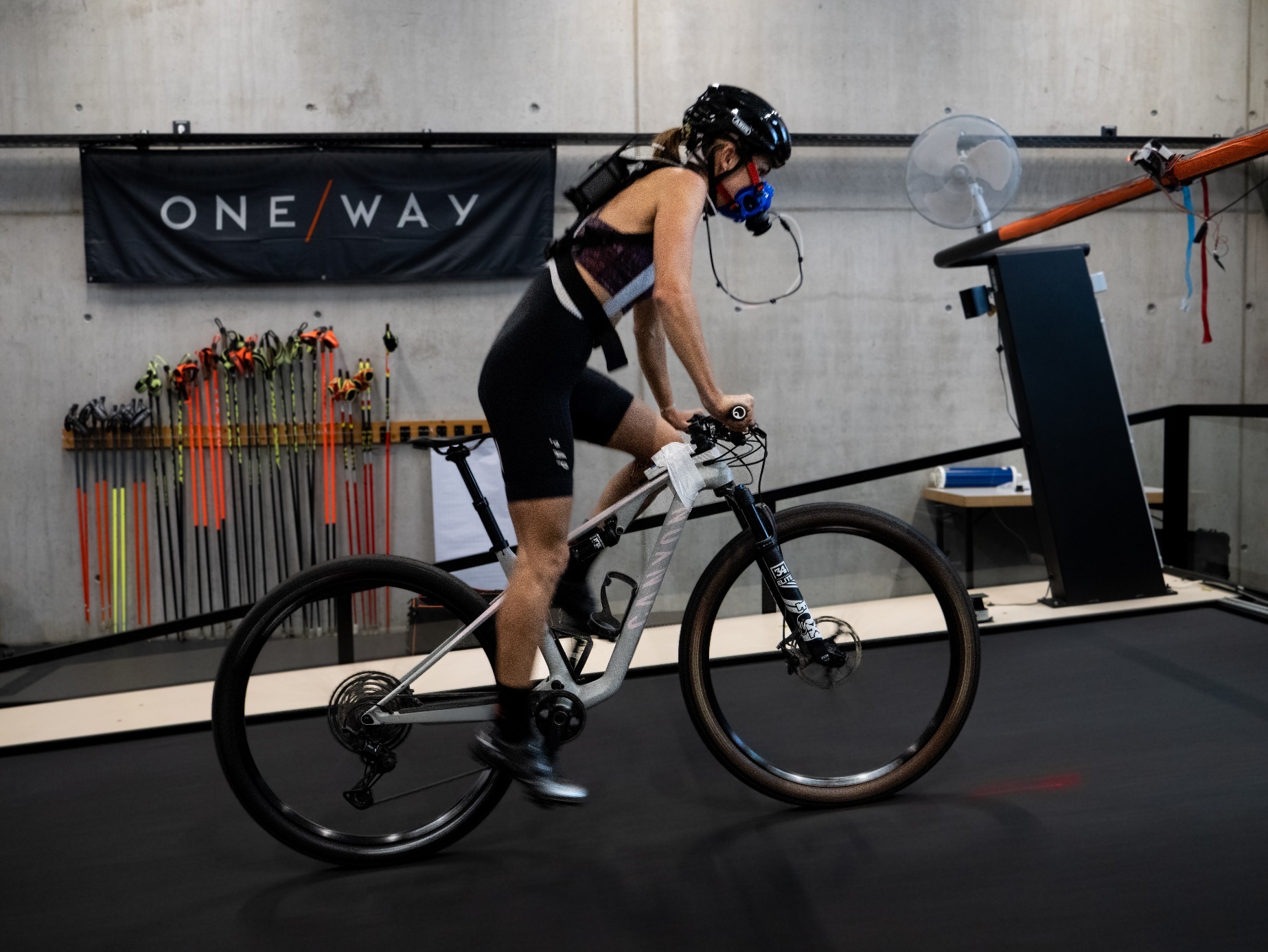


**Appendix 6:** Electronic circuit diagram for accelerometer used to capture lateral displacement.
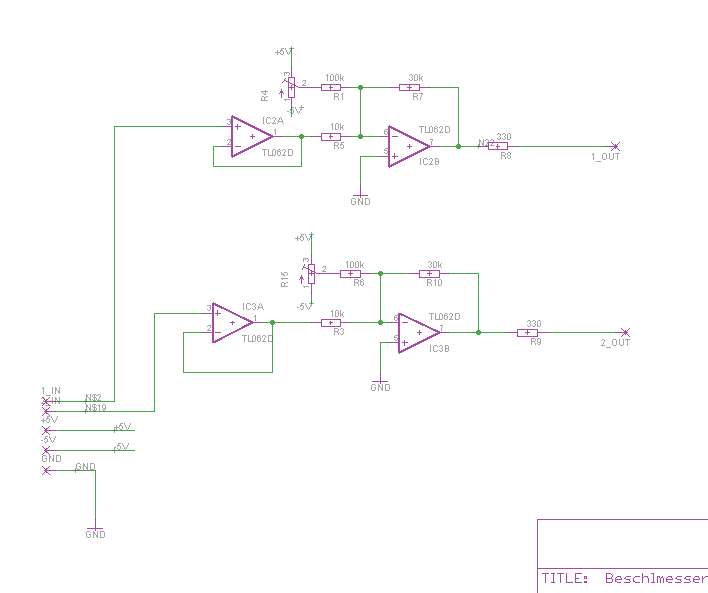


**Appendix 7:** Exercise protocols for the two interventions.

Abbreviations: HR = Heartrate

| Group | Week | Warm-up | Exercise | Sets | Repetitions | Progression |
| --- | --- | --- | --- | --- | --- | --- |
| **Trunk Muscle Strength** | 1-8 | 10 min jogging at 65-70% HR max | Lateral Cable Woodchopper crunch Pullup Bar windshield wipers Back extension machine Abdominis machine Medine ball L + R crunches | 3L & 3R 3 3 3 3 | 10 10 10 10 10 | Increase load weekly |
| Group | Week | Warm-up | Exercise | Sets | Repetitions | Progression |
| **Active Control** | 1-8 | 10 min jogging at 65-70% HR max | Squat jumps Counter movement jumps Barbell squat Barbell back squat Deadlift | 3 3 3 3 3 | 4 4 8 8 8 | Increase load weekly |

**
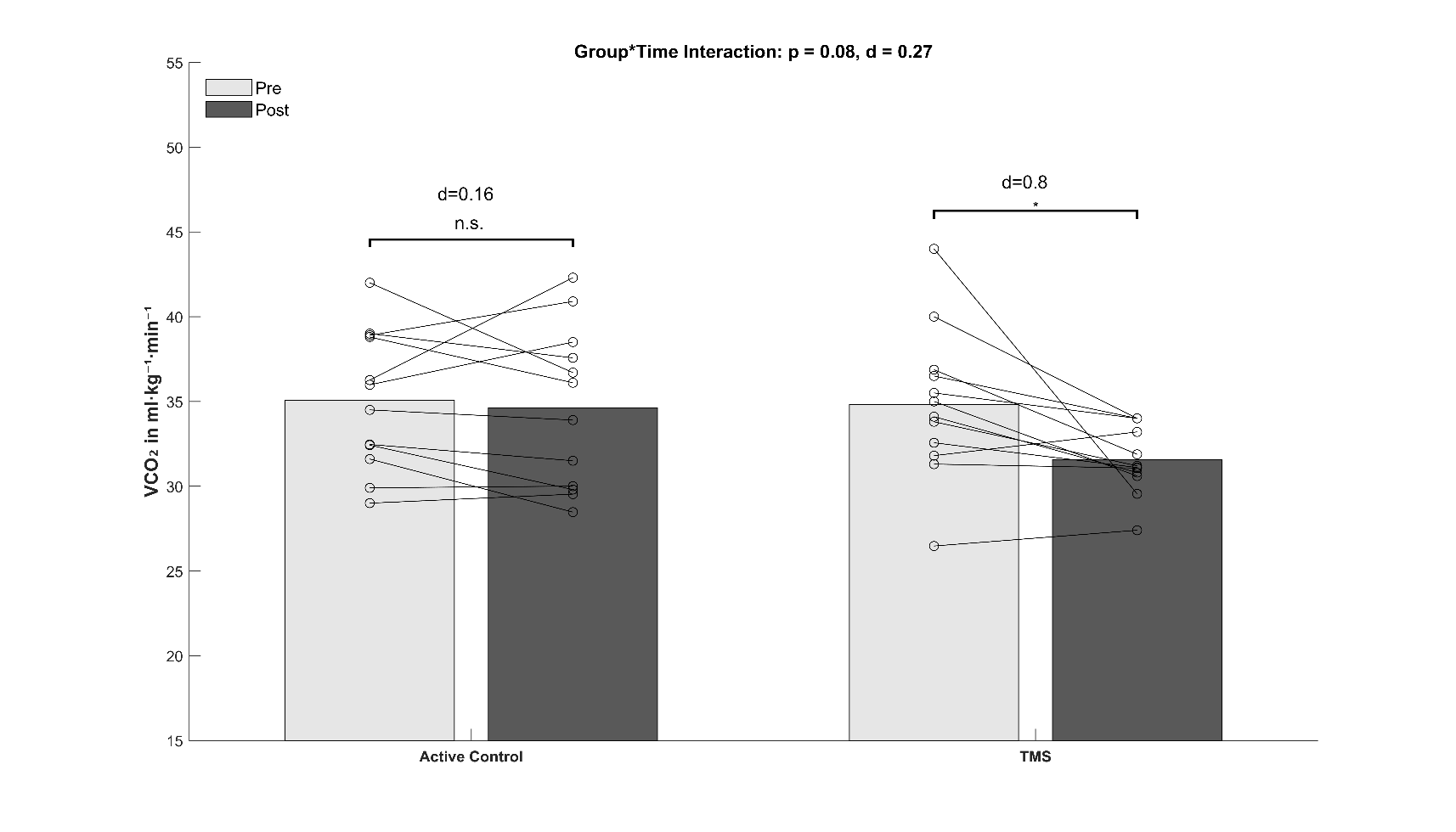
Appendix 8: Figures and tables for non-significant group by time interactions**

**Figure 6.** Mean values (bars) and individual data (lines) for V̇CO₂

Legend: d, Cohen’s d; * = p ­­< 0.05; ** = p < 0.005.

Abbreviations: TMS = trunk muscle strength group; V̇CO₂ = carbon dioxide output


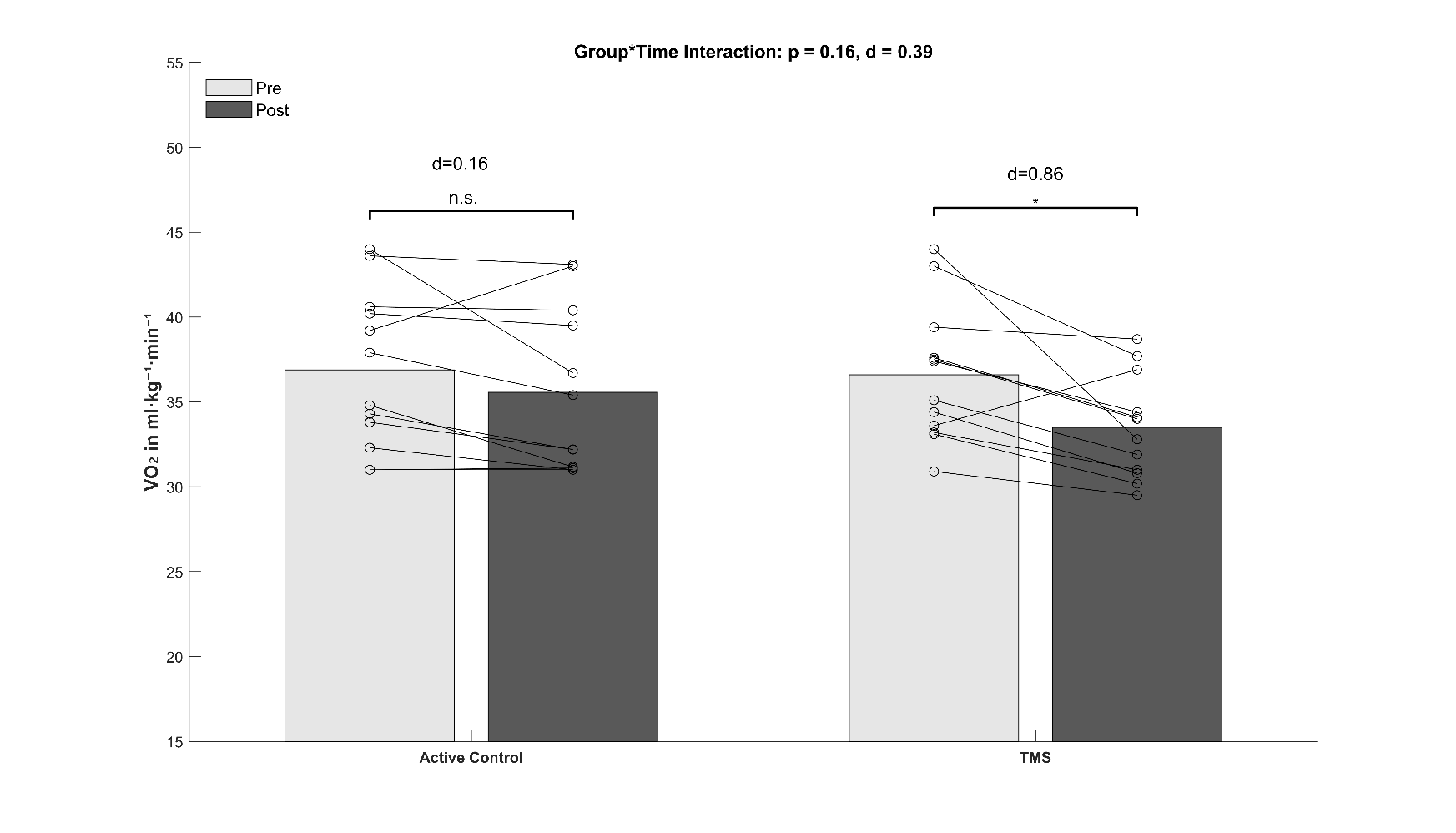


**Figure 7.** Mean values (bars) and individual data (lines) for V̇O₂

Legend: d, Cohen’s d; * = p ­­< 0.05; ** = p < 0.005.

Abbreviations: TMS = trunk muscle strength group; V̇O₂ = oxygen uptake


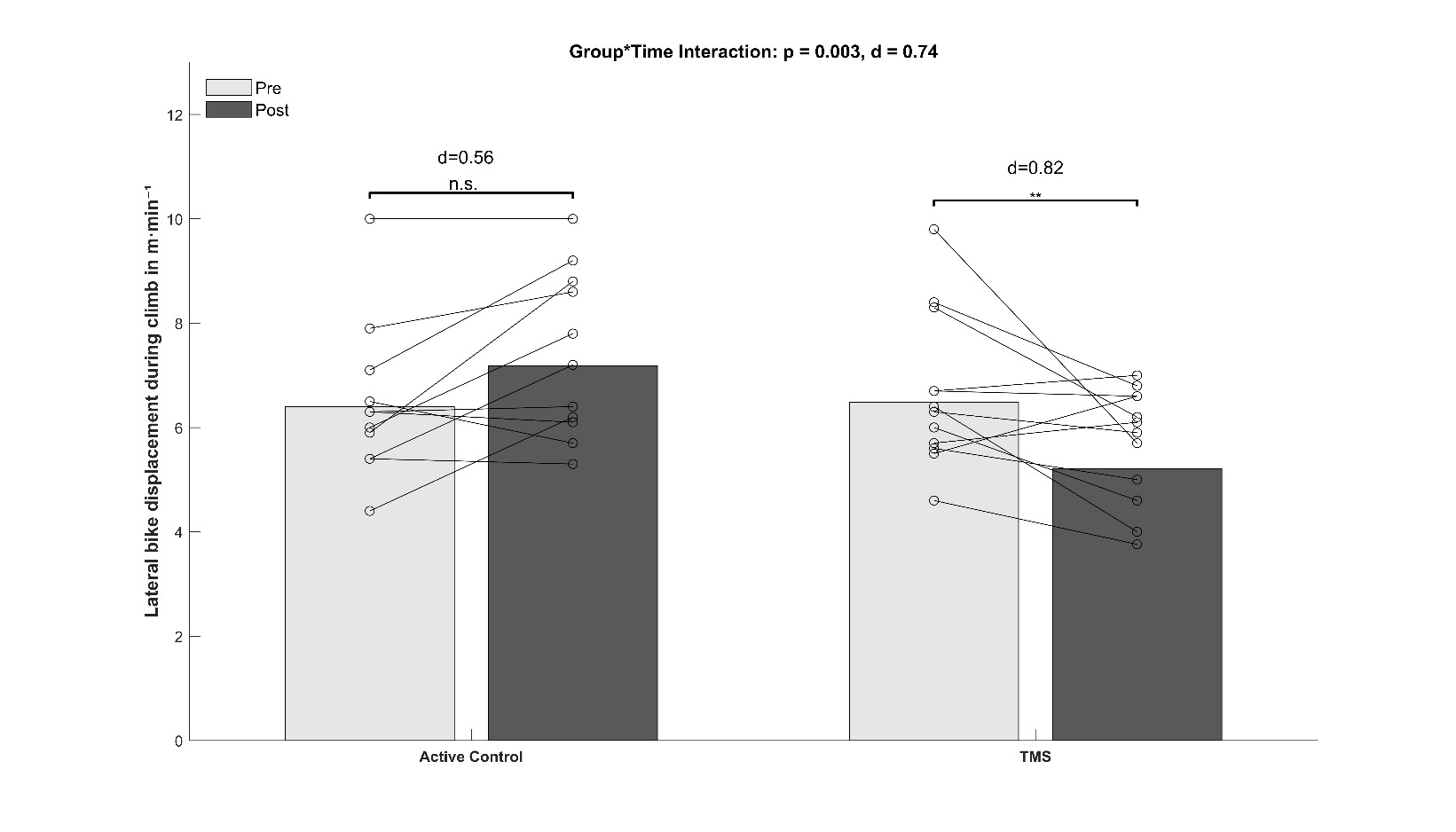


**Figure 8.** Mean values (bars) and individual data (lines) for lateral bike displacement during climbing

Legend: d, Cohen’s d; * = p ­­< 0.05; ** = p < 0.005.

Abbreviations: TMS = trunk muscle strength group

**Table 7.** Group-specific mean values and standard deviations for lateral displacement during climbing from pre to post (p-values and effect sizes [Cohen’s d]).

Abbreviations: TMS = Trunk muscle strength training group

|  | **Active Control** |  |  |  | **Trunk Muscle Strength** |  |  |  |  |  |  |
| --- | --- | --- | --- | --- | --- | --- | --- | --- | --- | --- | --- |
|  |  |  |  |  |  |  |  |  | Main Effect of **Group** | Main Effect of **Time** | Interaction of **Group*Time** |
| **Variable** | **PRE** | **POST** | **Δ %** | **95% CI** | **PRE** | **POST** | **Δ %** | **95% CI** | (p-value, Cohen's d) | (p-value, Cohen's d) | (p-value, Cohen's d) |
| **Lateral displacement climbing** | 6.47 ± 1.48 | 7.39 ± 1.58 | 14.2 % | 5.86 - 8.06 | 6.66 ± 1.50 | 5.68 ± 1.1 | -14.7% | 5.21 - 7.29 | 0.16 (0.31) | 0.92 (0.03) | 0.003 (0.74) |
| [m·min⁻¹] |  |  |  |  |  |  |  |  |  |  |  |
